# Improving mitochondria and ER stability helps eliminate upper motor neuron degeneration that occurs due to mSOD1 toxicity and TDP‐43 pathology

**DOI:** 10.1002/ctm2.336

**Published:** 2021-02-22

**Authors:** Barış Genç, Mukesh Gautam, Öge Gözütok, Ina Dervishi, Santana Sanchez, Gashaw M. Goshu, Nuran Koçak, Edward Xie, Richard B. Silverman, P. Hande Özdinler

**Affiliations:** ^1^ Department of Neurology, Feinberg School of Medicine Northwestern University Chicago Illinois USA; ^2^ Department of Chemistry Northwestern University Evanston Illinois USA; ^3^ Department of Molecular Biosciences, Chemistry of Life Processes Institute, Center for Molecular Innovation and Drug Discovery, Center for Developmental Therapeutics Northwestern University Evanston Illinois USA; ^4^ Department of Pharmacology, Feinberg School of Medicine Northwestern University Chicago Illinois USA; ^5^ Chemistry of Life Processes Institute Northwestern University Evanston IL 60208; ^6^ Mesulam Center for Cognitive Neurology and Alzheimer's Disease Northwestern University, Feinberg School of Medicine Chicago IL 60611; ^7^ Les Turner ALS Center Northwestern University, Feinberg School of Medicine Chicago IL 60611

**Keywords:** ALS, HSP, mSOD1, NU‐9, PLS, TDP‐43 pathology, upper motor neurons

## Abstract

**Background:**

Upper motor neurons (UMNs) are a key component of motor neuron circuitry. Their degeneration is a hallmark for diseases, such as hereditary spastic paraplegia (HSP), primary lateral sclerosis (PLS), and amyotrophic lateral sclerosis (ALS). Currently there are no preclinical assays investigating cellular responses of UMNs to compound treatment, even for diseases of the UMNs. The basis of UMN vulnerability is not fully understood, and no compound has yet been identified to improve the health of diseased UMNs: two major roadblocks for building effective treatment strategies.

**Methods:**

Novel UMN reporter models, in which UMNs that are diseased because of misfolded superoxide dismutase protein (mSOD1) toxicity and TDP‐43 pathology are labeled with eGFP expression, allow direct assessment of UMN response to compound treatment. Electron microscopy reveals very precise aspects of endoplasmic reticulum (ER) and mitochondrial damage. Administration of NU‐9, a compound initially identified based on its ability to reduce mSOD1 toxicity, has profound impact on improving the health and stability of UMNs, as identified by detailed cellular and ultrastructural analyses.

**Results:**

Problems with mitochondria and ER are conserved in diseased UMNs among different species. NU‐9 has drug‐like pharmacokinetic properties. It lacks toxicity and crosses the blood brain barrier. NU‐9 improves the structural integrity of mitochondria and ER, reduces levels of mSOD1, stabilizes degenerating UMN apical dendrites, improves motor behavior measured by the hanging wire test, and eliminates ongoing degeneration of UMNs that become diseased both because of mSOD1 toxicity and TDP‐43 pathology, two distinct and important overarching causes of motor neuron degeneration.

**Conclusions:**

Mechanism‐focused and cell‐based drug discovery approaches not only addressed key cellular defects responsible for UMN loss, but also identified NU‐9, the first compound to improve the health of diseased UMNs, neurons that degenerate in ALS, HSP, PLS, and ALS/FTLD patients.

## Graphical abstract

• Mitochondrial defects and endoplasmic reticulum (ER) disintegration are common causes of upper motor neuron (UMN) degeneration observed in the UMNs of both patients and mouse models.

• NU‐9, a novel compound with drug‐like properties, improves integrity of mitochondria and ER, as well as the stability of UMN apical dendrites.

• NU‐9 treatment eliminates ongoing degeneration of UMNs that become diseased due to mSOD1 toxicity and TDP‐43 pathology with behavioral outcomes.

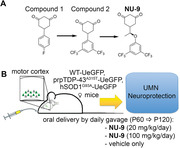

**Abbreviations**
ALS: amyotrophic lateral sclerosis
EM: electron microscopy
ER: endoplasmic reticulum
fALS: familial ALS
FTLD: frontotemporal lobar degeneration
GFP: green fluorescent protein
HSP: hereditary spastic paraplegia
mSOD1: misfolded superoxide dismutase protein
P: postnatal day
PLS: primary lateral sclerosis
sALS: sporadic ALS
UMN: upper motor neuron
WT: wild type

## 1 BACKGROUND

The corticospinal motor neurons (CSMN, a.k.a. upper motor neurons [UMNs]) have a unique role to collect, integrate, and transmit cerebral cortex's input to spinal cord targets, so that voluntary movement becomes the distilled end‐results of cortical input.^1^ UMNs are a clinically important and relevant neuron population, both within the context of injury and neurodegenerative diseases.^2^, ^3^, ^4^ Long‐term paralysis occur when UMNs degenerate in spinal cord injury patients.^5^ In diseases such as hereditary spastic paraplegia (HSP), primary lateral sclerosis (PLS), and amyotrophic lateral sclerosis (ALS), the identification, characterization, and manifestation of disease are considered as a function of UMN degeneration over time.^6^, ^7^, ^8^, ^9^, ^10^, ^11^ Therefore, improving the health of degenerating UMNs will have broad implications both within the context of injury and neurodegeneration.

The relevance of UMNs, especially with respect to ALS disease pathology, is becoming more evident, and the need to improve UMN health for developing effective and long‐term treatment strategies is now considered a necessity.^12^, ^13^ However, to date, there has been no compound and no effective treatment strategy that targets the health of UMNs. Interestingly, even for motor neuron diseases characterized by the progressive loss of UMNs, none of the compounds that moved into clinical trials has ever been tested for their ability to improve UMN health. Current preclinical *in vitro* tests involve a different set of cell lines, at times not related to motor neuron biology. Most preclinical *in vivo* studies utilize mouse models that are generated by the mutation or pathology detected in patients, and that closely recapitulate many of the human condition.^14^, ^15^, ^16^, ^17^, ^18^ So far, extension of lifespan in mouse models fails to translate to improved survival in patients^19^, ^20^, ^21^ and calls for better and more informative preclinical assessments that translate to the human disease condition. Even though motor neuron diseases develop mostly because motor neurons degenerate, there has never been a study that investigates the health and betterment of diseased UMNs at a cellular level.

The only two drugs that have been approved by the FDA to treat ALS are riluzole, approved in 1995, and edaravone, approved in 2017; the latter works as a free radical scavenger and has been previously prescribed for stroke patients.^22^, ^23^, ^24^, ^25^, ^26^, ^27^ The ability of edaravone to improve UMN health has not been tested, and its efficacy has been studied only with the superoxide dismutase protein (SOD1), and not on the TDP‐43, mouse model. Limited information is available on the cellular events that contribute to improved motor neuron survival.^22^, ^28^ Riluzole was approved prior to the development of hSOD1^G93A^ mice, and it works mainly on astrocytes to reduce astrogliosis‐mediated toxicity.^29^ Because riluzole failed to improve the longevity of the misfolded SOD1 (mSOD1) mouse model,^30^, ^31^ it probably would have failed preclinical testing had it been developed after the generation of hSOD1^G93A^ mice.

We and others find that UMNs in mice and UMNs in humans share many common features of motor neuron biology and display identical characteristics of neuropathology at the cellular level.^8^, ^32^, ^33^, ^34^, ^35^ For example, diseased UMNs in mice display degenerating apical dendrites, which is also observed in the UMNs of a broad spectrum of ALS patients, including sporadic ALS (sALS), familial ALS (fALS), and ALS/frontotemporal lobar degeneration (FTLD).^8^, ^35^, ^36^, ^37^ Likewise, the UMNs that become diseased due to TDP‐43 pathology have profound defects in their mitochondria and endoplasmic reticulum (ER), which are also observed in the UMNs of ALS patients with TDP‐43 pathology.^34^, ^38^ This important translation at the cellular level further suggests that the emphasis needs to be on the neurons that degenerate and that a mechanism‐focused and cell‐based preclinical drug discovery platform would be informative and translational.^39^, ^40^, ^41^ Furthermore, drug companies and the FDA now demand more information on the efficacy of compounds at the cellular level, which would expedite the success rate of clinical trials.

Misfolded SOD1 toxicity and TDP‐43 pathology represent two distinct, and mostly nonoverlapping, causes of ALS. Recent studies have reported a positive effect of small molecules on SOD1 and TDP‐43 models *in vivo*, but failed to investigate their impact on UMNs.^42^, ^43^, ^44^, ^45^ TDP‐43 pathology is mostly excluded from the brains of patients with SOD1 mutations, and misfolded SOD1 is not observed in cases with TDP‐43 pathology.^46^, ^47^, ^48^, ^49^, ^50^, ^51^ Therefore, being able to identify a compound that improves the health and stability of UMNs that become diseased due to these two different causes would have implications for a broad spectrum of patients.

Here we report that NU‐9, a compound that was previously identified based on its ability to reduce mSOD1 aggregation in cell lines, to cross the blood brain barrier, have low toxicity and favorable drug‐like properties,^52^, ^53^, ^54^, ^55^, ^56^ has profound efficacy on stabilizing the cellular integrity of UMNs that degenerate due to mSOD1 toxicity and TDP‐43 pathology. NU‐9 treatment restored the structural integrity of mitochondria and ER, improved cytoarchitectural stability and integrity of UMN apical dendrite, eliminated the ongoing UMN degeneration that occurs due to two distinct underlying causes, and improved motor function that is related to UMN health. This is the first mechanism‐focused and cell‐based drug discovery study that lays the foundation for studies that will identify compounds based on their ability to restore neuron health, and also reports NU‐9 as the first compound that eliminates UMN degeneration that occurs due to mSOD1 toxicity and TDP‐43 pathology, an important step in drug discovery efforts for ALS, HSP, PLS, and ALS/FTLD patients.

## 2 METHODS

### 2.1 Postmortem human brain samples

Postmortem human tissue collected according to protocols approved by an institutional review board was obtained from Northwestern University and University of Chicago. Clinical records were available for every subject. A neurologist examined all the patients and a neuropathologist had expertise in neurodegenerative disorders. Brains were fixed either in 10% neutral buffered formalin for 2 weeks or 4% paraformaldehyde (PFA) at 4°C for 30 h. Areas of the primary motor cortex were retrieved by an expert neuropathologist, and 70‐nm ultrathin sections were used for electron microscopy (EM) analysis, as previously described.^34^ In this study, motor cortex isolated from normal control subjects with no neurologic disease (*n* = 4) and ALS patients (*n* = 9) were included (Table S1).

### 2.2 Mice

All animal procedures were approved by the Northwestern University Animal Care and Use committee and complied with the standards of the National Institutes of Health. All mice were on C57BL/6 background. Transgenic hemizygous males expressing a high copy number of the human SOD1 gene with a *G93A* mutation (B6SJL‐Tg(SOD1*G93A)1Gur/J; The Jackson Laboratory) were bred to hemizygous UCHL1‐eGFP(green fluorescent protein) females to generate hSOD1^G93A^‐UeGFP and wild type (WT)‐UeGFP (control) mice. UCHL1‐eGFP mice were generated in the Ozdinler Lab; they are reporter lines for UMNs,^57^ and are now available at Jackson Laboratory (stock no. 022476). Hemizygous UCHL1‐eGFP females were bred to hemizygous prpTDP‐43^A315T^ mice (procured from Jackson Laboratory, stock no. 010700) to generate prpTDP‐43^A315T^‐UeGFP mice. In this study, only female mice were used for experiments and male mice were bred with WT females to generate more hSOD1^G93A^‐UeGFP or prpTDP‐43^A315T^ mice. prpTDP‐43^A315T^ mice were supplied with gel diet (DietGel 76A, ClearH_2_O, ME, USA) to eliminate gastrointestinal (GI) complications. Transgenic mice were identified by PCR amplification of DNA extracted from their tail, as previously described.^34^, ^57^, ^58^, ^59^

### 2.3 NU‐9 preparation and delivery

NU‐9 was prepared as described previously.^54^ Pharmacokinetic properties of NU‐9 are listed in Table 1. For the formulation of 10 mg/ml concentration, 36.58 mg of test compound NU‐9 was weighed, 0.274 ml of *N*‐methyl‐2‐pyrrolidone (NMP, Sigma‐Aldrich) was added and vortexed, then 3.384 ml of olive oil was added and vortexed for ∼2 min until a clear yellow formulation was obtained. The 20 and 100 mg/ml doses were prepared separately and stored.

**TABLE 1:** Pharmacokinetic (PK) properties of NU‐9

ADME/PK properties	NU‐9
MW	368.28
clogP	2.99
TPSA	43.37
Ligand efficiency	0.374
Rotatable bonds	3
H‐bond donors	0
H‐bond acceptors	3
EC_50_ (protein aggregation/toxicity)	300 nM
Solubility (aqueous)	≥100 µM (37 µg/ml)
Microsome stability (*t*_1/2_)	74 min (human) 52 min (mouse)
Plasma stability (*t*_1/2_)	75% remaining after 2 h @ 37°C
Plasma protein binding	90%
Caco‐2	A→B 24.1 × 10^–6^ cm/s B→A 1.5 × 10^–6^ cm/s Efflux ratio 0.06
PAMPA‐BBB	*P*_e_ 8.11 × 10^–6^ cm/s (CNS+)
hERG antagonism	No inhibition up to 30 µM
CYP inhibition	5 CYPs; <10% at 3 µM
Oral bioavailability	94%
*t*_1/2_	2.73 h
CL (mouse)	44 ml/min/kg
AUC	3452 h‐ng/ml po
Brain penetration	8.3 µM
Blood levels (*t*_max_) ip, mice (dose)	12 h (500 mg/kg)
MTD (mice, ip)	1280 mg/kg
NOAEL (mice, po)	100 mg/kg
SOD1 model mouse life extension	13% at 20 mg/kg

*Note*. NU‐9 was first identified for its ability to reduce mSOD1‐mediated toxicity.^52^ It has drug‐like and favorable pharmacokinetic properties.^53^, ^54^

Abbreviations: ADME, absorption, distribution, metabolism, and excretion; NOAEL, no observed adverse effect level.

hSOD1^G93A^‐UeGFP, prpTDP‐43^A315T^‐UeGFP and WT‐UeGFP mice were weighed and the required NU‐9 dose per weight was calculated (20 or 100 mg/kg), which was administered once daily by oral gavage, starting at postnatal day (P)60 and continuing until P120. Animals from the untreated group received the vehicle (NMP and olive oil) only. For oral administration purposes, 20 ga × 38 mm plastic feeding tubes were used (Instech Laboratories, Inc). The gavage tip was inserted into the mouth directly over the tongue and into the pharynx. While observing the swallowing reflex, the tip was safely and smoothly slid into the esophagus. Once the administration was completed, the gavage tip was pulled straight out.

In this study, WT‐UeGFP (*n* = 10) and hSOD1^G93A^‐UeGFP mice (*n* = 6) were treated with vehicle, WT‐UeGFP (*n* = 5) and hSOD1^G93A^‐UeGFP mice (*n* = 7) were treated with 20 mg/kg/day dosage of NU‐9, and WT‐UeGFP (*n* = 11), hSOD1^G93A^‐UeGFP (*n* = 9), and prpTDP‐43^A315T^‐UeGFP mice (*n* = 4) were treated with 100 mg/kg/day dosage of NU‐9. prpTDP‐43^A315T^‐UeGFP mice (*n* = 3), which received no treatment, were used as controls for the TDP‐43^A315T^ group (Table S2). Same animals were used for both immunofluorescence and EM analysis whenever possible.

### 2.4 Behavioral analyses

Behavior data were collected from hSOD1^G93A^‐UeGFP and WT‐UeGFP mice (starting at P60 and every 7 days until P116) and from prpTDP‐43^A315T^‐UeGFP mice (at P60, P90, and P120) with the following number of mice for each group: WT‐UeGFP vehicle (*n* = 10), hSOD1^G93A^‐UeGFP vehicle (*n* = 6), WT‐UeGFP 20 mg/kg/day NU‐9 (*n* = 5), hSOD1^G93A^‐UeGFP 20 mg/kg/day NU‐9 (*n* = 7), WT‐UeGFP 100 mg/kg/day NU‐9 (*n* = 11), hSOD1^G93A^‐UeGFP 100 mg/kg/day NU‐9 (*n* = 9), prpTDP‐43^A315T^‐UeGFP untreated (*n* ≥ 3), and prpTDP‐43^A315T^‐UeGFP 100 mg/kg/day NU‐9 (*n* = 4) (Table S3).

#### 2.4.1 Rotarod test

Mice were placed on a rotating rod that accelerates linearly from 4 to 40 rpm (Rotarod, Ugo Basile), and the average time spent on the rotating rod for three consecutive trials was calculated for each mouse. Mice were allowed to run for a maximum of 5 min with a 5 min rest period between each run, and the latency to fall was recorded for three consecutive trials. The average of the three trials was taken as the data point for each mouse at the age tested, and the performance of different groups are reported as the mean ± SEM.

#### 2.4.2 Hanging wire test

Mice were placed on a wire mesh, which was then inverted and suspended above the home cage; the time when the animal fell was recorded. This test was performed three times for a maximum of 60 s each session, with a 1‐min rest period between each trial. The average performance for each session is presented as the average of the three trials.

### 2.5 Histology

Mice were deeply anesthetized using ketamine (90 mg/kg) with xylazine (10 mg/kg), and transcardially perfused with 4% PFA in PBS. The brains were removed intact and postfixed (4% PFA, overnight) and stored in PBS with sodium azide (0.01%) at 4°C. Sections were cut in a coronal (50 µm) plane using a vibratome (Leica) and processed for immunocytochemical analyses.

### 2.6 Immunocytochemistry

The antibodies used are as follows: anti‐GFP (1:1000, Invitrogen; or 1:1000, Abcam) and anti‐misfolded SOD1 (B8H10, 1:250, Médimabs). Briefly, sections were treated with blocking solution (PBS, 0.05% BSA, 2% FBS, 1% Triton X‐100, and 0.1% saponin) for 30 min at room temperature and incubated with primary antibody diluted in blocking solution overnight at 4°C. Appropriate secondary fluorescent antibodies (1:500, AlexaFluor‐conjugated, Invitrogen) were added to the blocking solution at room temperature for 2 h in the dark.

### 2.7 UMN quantification

Since UMNs are genetically labeled with eGFP expression in the motor cortex of WT‐UeGFP, hSOD1^G93A^‐UeGFP, and prpTDP‐43^A315T^‐UeGFP mice, UMNs were identified based on the GFP expression. Quantitative analyses were performed on three matched rostrocaudal sections spanning the motor cortex. Three images per subject were taken to capture a 4× objective filed that encompass layer 5 of the motor cortex. An inverted epifluorescent Eclipse TE2000‐E microscope (Nikon) with the same exposure time and intensity was used. UMNs were counted only if their soma and apical dendrite were both visualized in the same 50‐µm thick section. Images were analyzed in ImageJ (NIH) using the Find maxima processing command to determine the local maxima within a predetermined region of interest that circumscribes the layer 5 of the motor cortex. A universal noise tolerance, which ignores the local maxima corresponding to background and autofluorescence, was applied to all images.

### 2.8 Lower motor neuron (LMN) quantification

A 5‐mm block of lumbar spinal cord was sectioned in 50‐µm thick sections with a vibratome (Leica), and every other section was used for immunohistochemical analysis. LMNs were identified based on their location in the ventral horn and expression of the molecular marker ChAT. 10× Images were captured using an inverted epifluorescent Eclipse TE2000‐E microscope (Nikon). GFP^+^/ChAT^+^ and NeuN^+^/ChAT^+^ LMNs were quantified per section. The average number of LMNs per section is reported per mouse with *n* = 5 mice per group.

### 2.9 Quantification of misfolded SOD1

50‐µm thick sections of primary motor cortex were captured using the 20× objective on an inverted epifluorescent Eclipse TE2000‐E microscope using the same settings and exposure time for all of the samples. eGFP positive (eGFP^+^) UMNs in focal plane with sharp boundaries and a visible nucleus were traced using ImageJ (NIH). ROI were transferred to the misfolded SOD1 channel, and the integrated density was measured to determine the levels of misfolded SOD1 in GFP^+^ UMNs only. Fifty to 90 UMNs were analyzed per mouse, and the average integrated density was reported per experimental group. For visualization purposes of misfolded SOD1 fluorescence intensity, nd2 files were opened in ImageJ and spectrum look‐up table (LUT) was applied to the appropriate channel.

### 2.10 Electron microscopy

Mice were perfused with EM grade 4% PFA. One hemisphere of the brain was sectioned at 50 µm thickness coronally on a vibratome (Leica VT1000S, Leica Inc., Nussloch, Germany). The sections were postfixed in 2% PFA and 0.5% glutaraldehyde for 1 h, they were cryoprotected with glycerol–dimethylsulfoxide (DMSO) mixture followed by freeze–thaw at least four times, and treated with 1% sodium borohydride. They were then treated with 0.3% H_2_O_2_–10% methanol in TBS (100 mM Tris–HCl and 150 mM NaCl, pH 7.6) and 5% normal goat serum–1% bovine serum albumin in TBS to block nonspecific binding of primary antibody. This mixture was incubated overnight with rat anti‐Ctip2 antibody (1:500, Thermo Fisher Scientific, Rockford, IL, USA). Biotinylated goat anti‐rat IgG (1:500, Vector Laboratories, Burlingame, CA, USA) was used as the secondary antibody, and diaminobenzidine (DAB) was applied as the chromogen (ABC Elite kit, Vector Laboratories, Burlingame, CA, USA). Sections were then postfixed in buffered 2% osmium tetroxide (OsO_4_) (Electron Microscopy Sciences, Hatfield, PA, USA), rinsed with distilled water, and stained in 1% uranyl acetate (Electron Microscopy Sciences, Hatfield, PA, USA), again rinsed with distilled water, dehydrated in ascending grades of ethanol with transition fluid propylene oxide (Electron Microscopy Sciences, Hatfield, PA, USA), embedded in the resin mixture with Embed 812 (Electron Microscopy Sciences, Hatfield, PA, USA), and cured in a 60°C oven for 3 days. The sections in which primary motor cortex was present and visible under bright‐field illumination on a dissecting scope were selected. Approximately, 5‐mm‐wide × 7‐mm‐long pieces of the motor cortex from these sections were dissected under the microscope mounted on a resin block and were sectioned on a Leica Ultracut UC6 ultramicrotome (Leica Inc., Nussloch, Germany). The motor cortex was further trimmed, and only layer 5 was kept intact for preparing ultrathin sections. UMNs were identified based on the presence of Ctip2 immunohistochemistry (DAB) in their nuclei (Figure S1). Seventy‐nanometer‐thin sections were collected on 200‐mesh copper–palladium grids. Grids were counterstained with 8% radioactive depleted uranyl acetate and 0.2% lead citrate. Grids were examined on FEI Tecnai Spirit G2 TEM (FEI company, Hillsboro, OR, USA), and digital images were captured on a FEI Eagle camera.

### 2.11 Quantification of mitochondria and ER

EM images of Betz cells of normal control and patients, Ctip2‐positive (Ctip2^+^) UMN of WT, and hSOD1^G93A^‐UeGFP and prpTDP‐43^A315T^‐UeGFP mice (Figure S1) were acquired from 70‐nm ultrathin sections of the motor cortex, and EM images were taken on FEI Tecnai Spirit G2 TEM using FEI Eagle camera. Mitochondria were quantified, as described previously.^34^ Mitochondria with intact outer and inner membrane and with intact cristae were considered healthy. Mitochondria with morphological defects, such as broken outer and/or inner membrane and cristae, were counted individually and were reported as percentage of defective mitochondria. ER that are disintegrated and that display profound expansion were considered unhealthy. Length of ER cisternae was measured using line tool of ImageJ software (NIH) upon calibration for pixels/micrometer using set scale function. Individual ER cisternae were traced using freehand line tool of ImageJ. The length of each traced cisternae per UMN per section was recorded. On an average, 8–10 cells/group were used for measurement of ER cisternae. The total number of mitochondria and ER, and the total number of UMNs investigated by EM‐based quantification are presented in Table S4.

### 2.12 Statistical analysis

All analyses were performed using Prism software (GraphPad Software). The D'Agostino and Pearson normality test was performed on all datasets. Statistical differences between two groups were determined using either a parametric (Student's *t*‐test) or a nonparametric test (Mann–Whitney *t*‐test), when appropriate. For pairwise comparison of two groups, an unpaired *t*‐test with Welch's correction was used. Statistical differences between more than two groups were determined by one‐way ANOVA, followed by the Tukey's post hoc multiple‐comparison test. Two‐way ANOVA with Tukey's post hoc multiple‐comparison test was used for comparison of behavior data of SOD1 mice, and mixed‐effects analysis with Tukey's multiple‐comparison test was used for TDP‐43 mice. Statistically significant differences were taken at *p* < .05. Please refer to Tables S3 and S5 for results of all statistical tests performed.

## 3 RESULTS

### 3.1 Diseased UMNs in patients and UMNs of disease models share common cellular defects

**FIGURE 1:**
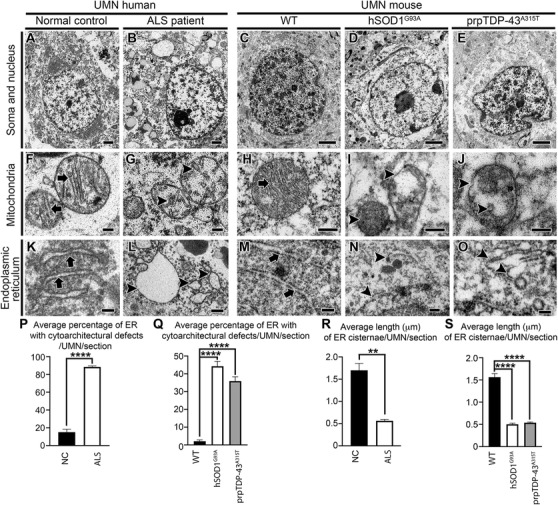
Upper motor neurons (UMNs) display ultrastructural defects in amyotrophic lateral sclerosis (ALS) patients, and in the mouse models that are diseased due to different underlying causes. (A) Representative electron microscopic (EM) image of UMN of normal control appears intact while (B) UMN of ALS patient showing cytoarchitectural defects. (C) EM image of UMN of WT mouse. (D) Representative EM image of UMN of hSOD1^G93A^, and (E) prpTDP‐43^A315T^ mouse displaying massive ultrastructural disintegration. (F) The mitochondria in a normal control showing intact inner mitochondrial membranes (arrows), as opposed to (G) mitochondria in ALS patient that displays disintegration of inner mitochondrial membrane (arrowheads). (H) Mitochondria in WT mouse appears to be structurally intact with distinct inner and outer mitochondrial membranes (arrow), whereas (I) mitochondria in UMN in a hSOD1^G93A^ and (J) prpTDP‐43^A315T^ mouse displaying severe disintegration of inner mitochondrial membranes (arrowheads). (K) Electron micrographs of UMN endoplasmic reticulum (ER) in a normal control display properly stacked long cisternae (arrows), but (L) ER in ALS patient shows distension and ballooning of ER cisternae (arrowheads). Similarly, (M) ER in a WT mouse (arrows) looks structurally intact in contrast to (N) ER in UMN of hSOD1^G93A^ and (O) prpTDP‐43^A315T^ mouse that displaying broken, short, and disintegrated ER cisternae (arrowheads). (P) Quantification of average percentage of ER with cytoarchitectural defects/UMN in ALS patients. ****p < .0001, Student's *t*‐test. (Q) Quantification of average percentage of ER with cytoarchitectural defects/UMN in hSOD1^G93A^. ****p < .0001, and prpTDP‐43^A315T^ mice. ****p < .0001, One‐way ANOVA followed by Tukey's post hoc multiple‐comparison test. (R) Quantification of average length of ER cisternae/UMN in ALS patients. **p < .001, Student's *t*‐test. (S) Quantification of average length of ER cisternae/UMN in hSOD1^G93A^ **** *p* < .0001, and prpTDP‐43^A315T^ mice. ****p < .0001, One‐way ANOVA followed by Tukey's post hoc multiple‐comparison test. Scale bars: A–E = 2 µm; F–O = 200 nm

UMN loss is a defining characteristic of ALS, and diseased UMNs display pathology at a cellular level (Figure 1).^8^, ^34^, ^35^ Likewise, the UMNs of hSOD1^G93A^ and the prpTDP‐43^A315T^ mice, which were developed to mimic mSOD1 toxicity and TDP‐43 pathology mediated motor neuron degeneration in patients,^58^, ^59^ develop progressive UMN loss^34^, ^57^, ^60^ and display similar defects at a cellular level. EM revealed such striking similarities between UMNs, albeit in different species (Figure 1).

Prior to processing for EM, mouse tissue sections were subjected to immunostaining for Ctip2, a marker for UMN^1^, ^36^, ^57^, ^61^ to distinguish UMN in layer 5 of the mouse motor cortex (Figure S1). UMN in human motor cortex were determined by their large soma size and deep layer 5 location, as previously described.^8^, ^34^ UMNs of normal control cases (*n* = 4) showed intact cell body with preserved cellular organelles (Figure 1A). Similarly, UMN of WT healthy mice showed well‐preserved soma and nucleus (Figure 1C). However, UMNs of ALS patients (*n* = 9) displayed massive ultrastructural defects especially at the site of mitochondria and ER (Figure 1B). The UMNs of both hSOD1^G93A^ (Figure 1D) and prpTDP‐43^A315T^ mice (Figure 1E) had similar problems.^34^

The ER in UMNs of normal controls and WT mice were well preserved with intact cisternae, adorned with ribosomes (Figure 1K, M arrows). Since smooth ER is very thin and difficult to identify accurately in tissue sections processed for immunoelectromicroscopic analysis, only rough ER with ribosomes were included in analyses. However, ER in UMNs of ALS patients were distended, swollen, and cisternae were damaged (Figure 1L arrowheads; normal control: 15% ± 3% ER cisternae/UMN/section; ALS: 88% ± 1% ER cisternae/UMN/section; *p* < .0001, Figure 1P). Same ultrastructural ER defects were also detected in the UMN of hSOD1^G93A^ (Figure 1N arrowheads; WT: 2% ± 1% cisternae/UMN/section; hSOD1^G93A^: 44% ± 3% ER cisternae/UMN/section; adjusted *p*‐value = .0001, Figure 1Q) and prpTDP‐43^A315T^ mice (Figure 1O arrowheads; Q; WT: 2% ± 1% ER cisternae/UMN/section; prpTDP‐43^A315T^: 36% ± 2% ER cisternae/UMN/section; adjusted *p*‐value = .0001). The average length of individual cisternae of ER was significantly shorter in UMNs of ALS cases compared to normal control (normal control: 1.7 ± 0.15 µm; ALS: 0.56 ± 0.03 µm; *p* < .001, Figure 1R). Similarly, as compared to WT, the average length of individual cisternae of ER was shorter in UMN of hSOD1^G93A^ (WT: 1.56 ± 0.07 µm; hSOD1^G93A^: 0.50 ± 0.02 µm; adjusted *p*‐value = .0001, Figure 1S), and prpTDP‐43^A315T^ mice (WT: 1.56 ± 0.07 µm; prpTDP‐43^A315T^: 0.53% ± 0.01%; adjusted *p*‐value = .0001, Figure 1S).

### 3.2 Identification of NU‐9

**FIGURE 2:**
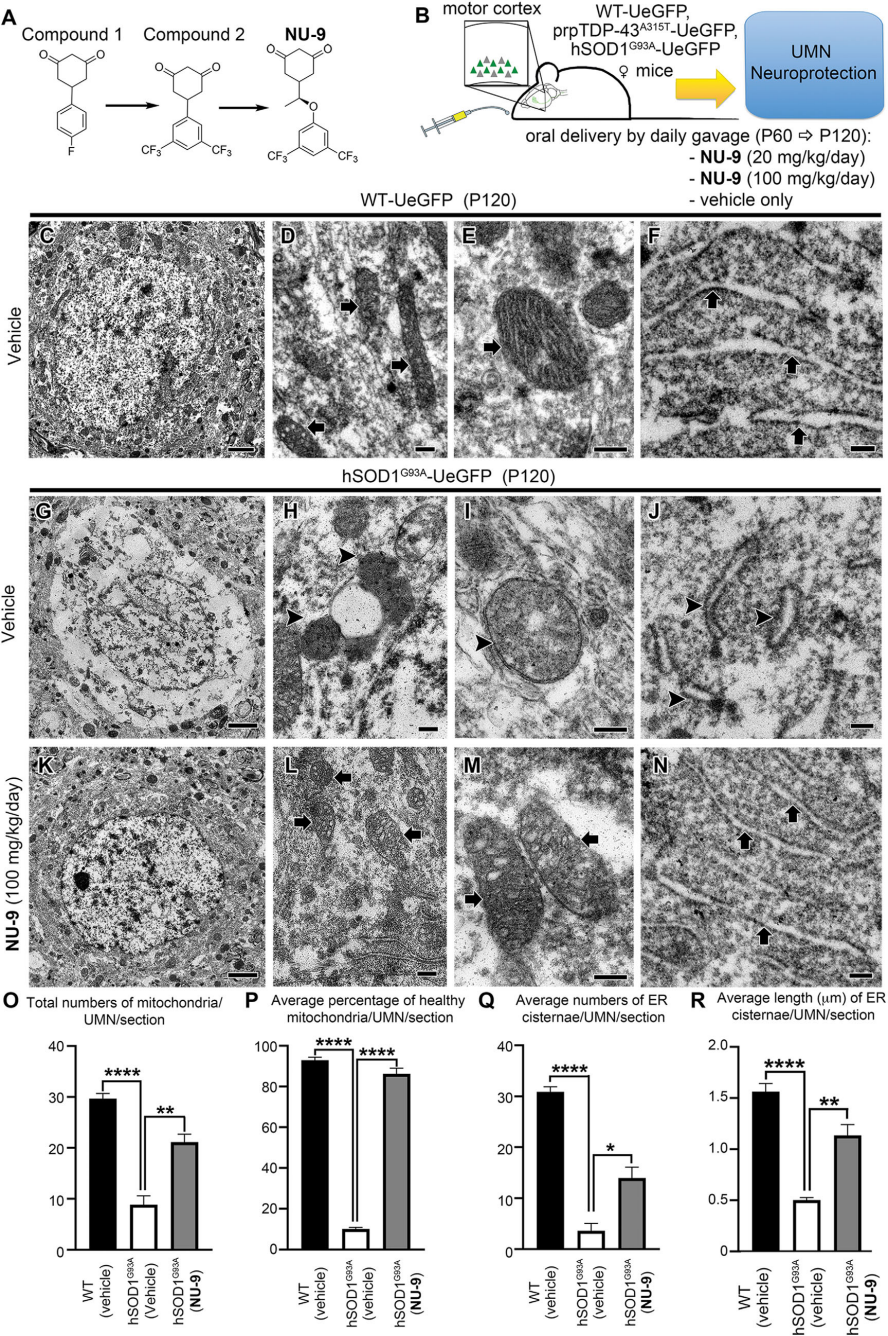
NU‐9 treatment improves ultrastructural integrity of both mitochondria and endoplasmic reticulum (ER) of upper motor neurons (UMNs) that become diseased from mutant SOD1 toxicity. (A) Progression from hit to lead to NU‐9 with chemical structure of NU‐9. (B) Experimental design for *in vivo* studies. (C–F) Representative electron microscopic (EM) images of UMNs in the motor cortex of WT‐UeGFP mice treated with vehicle at P120. Arrows point to mitochondria with intact inner membrane and ER with intact cisternae. Scale bars, C: 2 µm; D–F: 200 nm. (G–J) Representative EM images of UMNs in the motor cortex of hSOD1^G93A^‐UeGFP mice treated with vehicle at P120. (G) The cytoplasm is mostly devoid of major key organelles, and (H) there are numerous electron dense aggregates (arrowheads) and large droplets. (I) Mitochondria that lost the integrity of its inner membrane (arrowhead) or have overall structural damage, and (J) fragmented pieces of the ER (arrowheads) are evident. Scale bars, G: 2 µm; H–J: 200 nm. (K–N) Representative EM images of UMNs in the motor cortex of hSOD1^G93A^‐UeGFP mice treated with 100 mg/kg/day NU‐9. (K) Overall improvement in the cytoplasm with the presence of numerous organelles. (L) Mitochondria appear to be healthy (arrows), and the cytoplasm lacks major dense aggregates and droplets. (M) The integrity of the inner mitochondrial membrane and cristae structure is restored (arrows), and (N) ER cisternae (arrows) are arranged in proper structure without any fragmentation. Scale bars, I: 2 µm; L–N: 200 nm. (O) Quantification of total number of mitochondria/UMN in hSOD1^G93A^‐UeGFP mice with NU‐9 treatment. **p < .006, One‐way ANOVA followed by Tukey's post hoc multiple‐comparison test. (P) Quantification of percentage of healthy mitochondria/UMN in hSOD1^G93A^‐UeGFP mice with NU‐9 treatment. ****p < .0001, One‐way ANOVA followed by Tukey's post hoc multiple‐comparison test. (Q) Quantification of the number of ER cisternae/UMN in hSOD1^G93A^‐UeGFP mice with NU‐9 treatment. *p < .016, One‐way ANOVA followed by Tukey's post hoc multiple‐comparison test. (R) Quantification of average length of ER cisternae/UMN in hSOD1^G93A^‐UeGFP mice with NU‐9 treatment. **p < .002, One‐way ANOVA followed by Tukey's post hoc multiple‐comparison test

A high‐throughput screen of >50,000 drug‐like compounds was carried out using a SOD1^G93A^‐expressing PC12 cell‐based assay^55^, ^56^ to identify neuron‐protection scaffolds that mitigated protein aggregation and cytotoxicity.^52^ The cytotoxicity protection assay identified compounds that protected cells from the toxicity of aggregated SOD1^G93A^, and the protein aggregation assay targeted compounds that inhibited SOD1^G93A^induced protein aggregation. Hits from three families of compounds were filtered by a ligand‐based computational approach, including substructure and similarity searching^62^ and a clustering technique.^63^ A hit compound **1** (Figure 2A) was selected from >50 analogs of the cyclohexane‐1,3‐dione family of compounds, based on its ability to reduce mSOD1‐mediated toxicity and to inhibit mSOD1‐induced protein aggregation in a PC12 cell‐based assay.^52^ Multiple rounds of optimization were carried out, resulting in compound **2** (Figure 2A), which also had excellent*in vitro* absorption, distribution, metabolism, and excretion (ADME) properties, but did not penetrate into neurons.^53^ Further modifications of compound **2**, led to the generation of NU‐9 (Figure 2A),^54^ which penetrated cortical neurons, crossed the blood brain barrier, and had favorable pharmacokinetic properties^54^ (Table 1). In summary, NU‐9 had good *in vitro* potency (300 nM), microsome stability (*t*_1/2_ 74 min), plasma stability (*t*_1/2_ >2 h), little inhibition of cytochrome P450s and the hERG channel, good brain permeability (8 µM), and extended the lifespan of the ALS mouse by 13% at 20 mg/kg. *In vivo* pharmacokinetics in mice showed *t*_1/2_ = 2.7 h and 94% oral bioavailability. A 7‐day repeat toxicology study in mice gave a no observed adverse effect level (NOAEL) of 100 mg/kg, indicating negligible levels of toxicity.

### 3.3 NU‐9 treatment improves the integrity of mitochondria and ER

We recently generated a reporter line for UMNs, UCHL1‐eGFP mice, in which UMNs are genetically labeled with eGFP expression that is stable and long lasting,^57^ so that their cellular responses to compound treatment can be quantitatively assessed both *in vitro* and *in vivo*.^34^, ^57^ In an effort to visualize diseased UMNs and to assess their cellular response to compound treatment, hSOD1^G93A^ ^59^ and the TDP‐43^A315T^ mice^58^ were crossed with UCHL1‐eGFP to generate UMN reporter disease models, hSOD1^G93A^‐UeGFP and prpTDP‐43^A315T^‐UeGFP mice, in which UMNs with mSOD1 toxicity and TDP‐43 pathology were labeled with eGFP expression.^57^ NU‐9 (Figure 2A) was delivered to both hSOD1^G93A^‐UeGFP and WT‐UeGFP mice (Figure 2B, Table S2) at two different doses (20 and 100 mg/kg/day) daily via oral gavage starting at P60, when mice begin to show symptoms and UMNs display cellular defects.^60^ All mice were sacrificed at P120, which is considered end stage and about 60% of UMNs in the motor cortex are lost while the remaining UMNs have smaller soma size and vacuolated and disintegrated apical dendrites.^57^

EM allowed cell type‐specific analyses of UMNs and their key organelles with high precision at the ultrastructural level (Figure 2C–F). At P120, UMNs of vehicle treated hSOD^G93A^‐UeGFP mice lost most of their cytoplasmic integrity. There were very few intact organelles in the soma (Figure 2G). However, the presence of disintegrated mitochondria (Figure 2H,I arrowheads) and ER (Figure 2J arrowheads) were strikingly evident. Healthy mitochondria were defined by the presence of double membranes, and intact cristae structure. Mitochondria mostly lost the integrity of their inner membrane (Figure 2I arrowhead), aggregated, enlarged, or began to disintegrate. The ER also displayed broken and dispersed cisternae (Figure 2J arrowheads). Such profound ultrastructural defects at an organelle level begin to reveal the cellular problems diseased UMNs face in hSOD^G93A^‐UeGFP mice at P120, and it is thus of great importance to investigate whether NU‐9 treatment would have an impact.

NU‐9 treatment displayed profound improvements in both the structure and integrity of mitochondria and ER of diseased UMNs (100 mg/kg day dose, the only dose investigated at the EM level; Figure 2K–N). Upon treatment, the overall picture of the soma was dramatically improved with the presence of an intact nuclear membrane, which was devoid of any invaginations or protrusions, and detection of numerous organelles that were proper in size, location, and interactions among each other (Figure 2K). The mitochondrial inner membrane was intact with proper cristae (Figure 2L,M arrows), which were in close contact with the ER (Figure 2N arrows). The integrity and structure of the ER were maintained, and the expansion of the lumen was eliminated (Figure 2N arrows).

We next performed quantitative analyses to investigate whether these improvements were widely observed in diseased UMNs treated with NU‐9 (Table S4). The total number of mitochondria in UMNs of hSOD^G93A^‐UeGFP mice significantly increased upon 100 mg/kg/day NU‐9 treatment (21 ± 2 mitochondria/UMN/section; adjusted *p*‐value = .001) when compared to UMNs treated with vehicle (9 ± 2 mitochondria/UMN/section). The total number of mitochondria after NU‐9 treatment were comparable to WT‐UeGFP mice (30 ± 1 mitochondria/UMN/section; adjusted *p*‐value = .07). Furthermore, NU‐9 treatment significantly increased the percentages of healthy mitochondria in UMNs of hSOD^G93A^‐UeGFP mice (WT‐UeGFP: 93% ± 1%; vehicle: 10% ± 1%; NU‐9 100 mg/kg/day: 86% ± 3%; adjusted *p*‐value = .0001; Figure 2O,P).

NU‐9 treatment also significantly improved the integrity of ER in diseased UMNs; there were significantly more healthy cisternae (WT‐UeGFP: 31 ± 1; hSOD^G93A^‐UeGFP vehicle: 4 ± 1 ER cisternae/UMN/section; hSOD^G93A^‐UeGFP 100 mg/kg/day NU‐9: 14 ± 2 ER cisternae/UMN/section, adjusted *p*‐value = .008; Figure 2Q). In addition, the average length of ER cisternae of hSOD^G93A^‐UeGFP UMN treated with NU‐9 (1.13 ± 0.1 µm) became comparable to the length of ER cisternae of WT‐UeGFP UMN (1.56 ± 0.07 µm; adjusted *p*‐value = .014), whereas diseased UMN had significantly shorter ER cisternae (0.50 ± 0.02 µm; adjusted *p*‐value = .0001; Figure 2R; please refer to Table S4 for a complete list of total numbers of UMNs investigated and the total number of mice used for each quantitative analysis).

### 3.4 NU‐9 treatment reduces mSOD1 in UMNs of hSOD1^G93A^‐UeGFP mice

**FIGURE 3:**
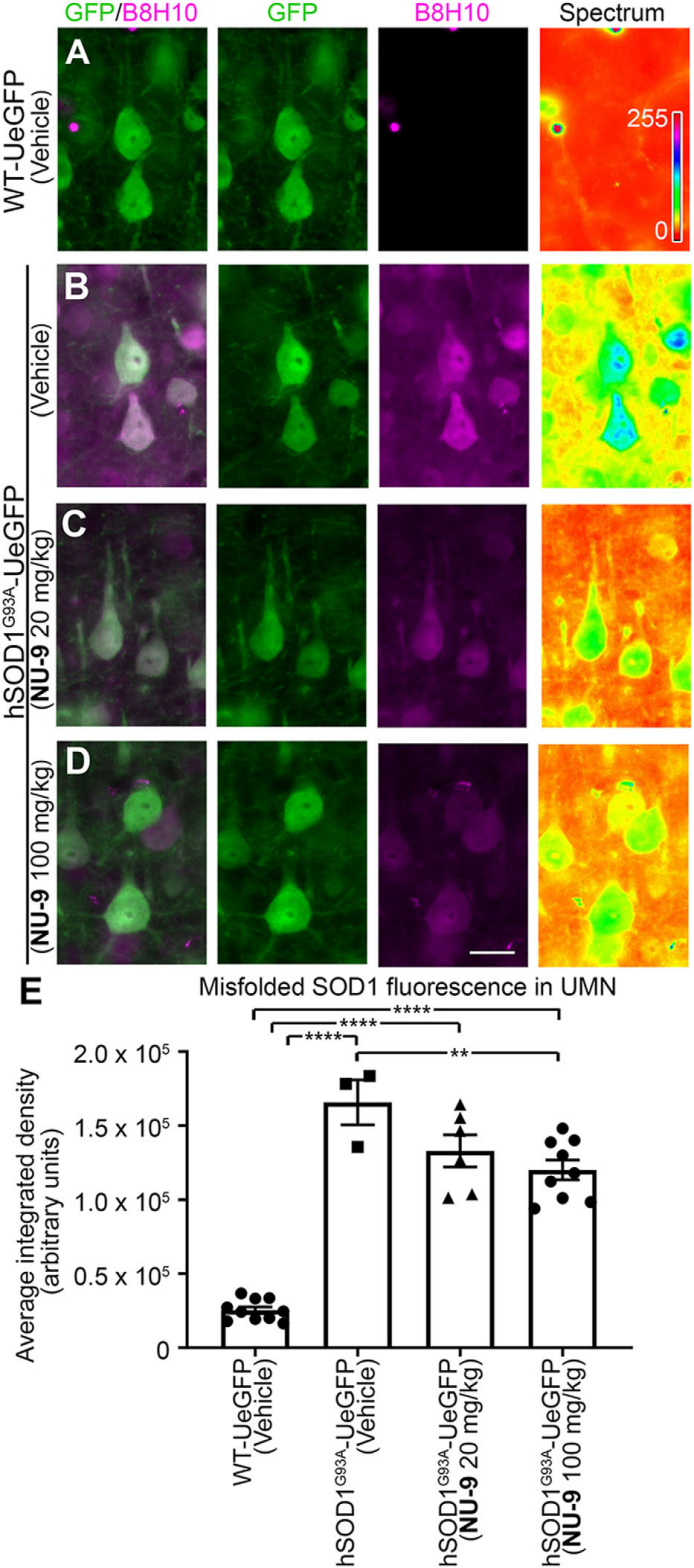
NU‐9 treatment reduces misfolded SOD1 levels in upper motor neurons (UMNs) of hSOD1^G93A^‐UeGFP mice. (A) Representative images of UMNs and B8H10 antibody staining that recognizes misfolded SOD1 protein in the motor cortex of WT‐UeGFP or (B) hSOD1^G93A^‐UeGFP mice treated with vehicle, (C) 20 mg/kg/day NU‐9, or (D) 100 mg/kg/day NU‐9. Scale bars, 20 µm; *n* ≥ 3 biological replicates. (E) Average integrated density of misfolded SOD1 fluorescence in UMNs in the motor cortex of WT‐UeGFP or hSOD1^G93A^‐UeGFP mice treated with vehicle, 20 mg/kg/day NU‐9, or 100 mg/kg/day NU‐9; mean, SEM, and individual data points shown for *n* ≥ 3 biological replicates. **p < .01, ****p < .0001, One‐way ANOVA followed by Tukey's post hoc multiple‐comparison test

NU‐9 was identified on the basis of its ability to reduce misfolded mSOD1 in PC12 cells.^55^, ^56^ Since UMNs of hSOD1^G93A^‐UeGFP mice contained misfolded SOD1,^64^ we next investigated whether NU‐9 treatment would reduce levels of mSOD1 in diseased UMNs. To investigate the presence of misfolded SOD1 protein, we used the well‐characterized B8H10 monoclonal antibody that can detect a wide spectrum of SOD1 mutants and metal‐depleted WT SOD1 protein, but not intact WT SOD1.^65^ UMNs in WT‐UeGFP mice do not have misfolded SOD1, as expected (Figure 3A, Figures S2 and S3). However, UMNs of hSOD1^G93A^‐UeGFP mice and UMNs of hSOD1^G93A^‐UeGFP mice treated with vehicle have high levels of misfolded SOD1 (1.66 × 10^5^ ± 0.15 × 10^5^ arbitrary units [au]; Figure 3B,E). NU‐9 treatment significantly reduces levels of mSOD1, especially in diseased UMNs (20 mg/kg/day: 1.33 × 10^5^ ± 0.11 × 10^5^ au; 100 mg/kg/day: 1.2 × 10^5^ ± 0.07 × 10^5^ au; adjusted *p*‐value = .007; Figure 3C–E).

### 3.5 NU‐9 treatment improves cytoarchitectural integrity of UMN apical dendrite

**FIGURE 4:**
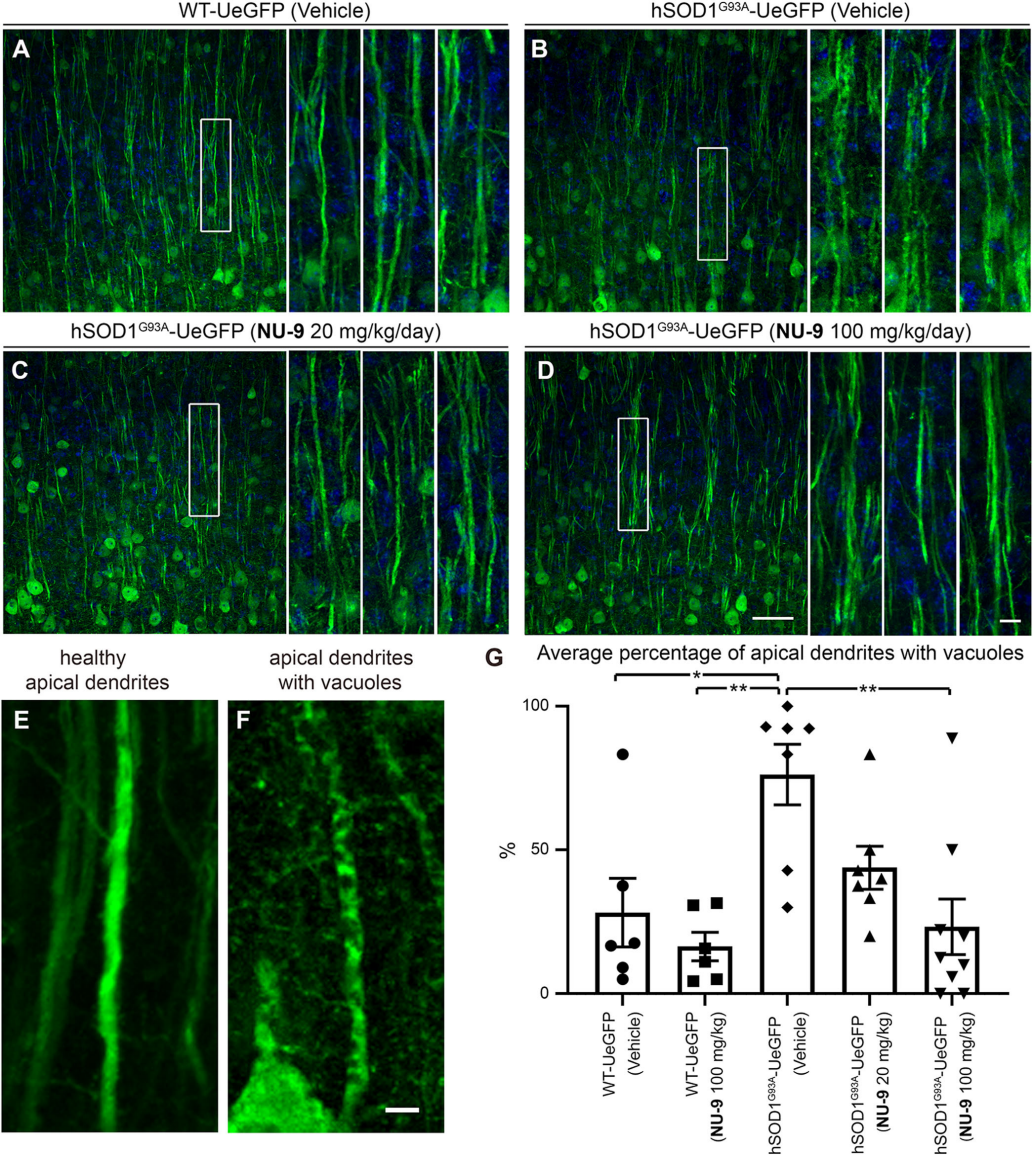
NU‐9 treatment improves cytoarchitectural integrity of disintegrating apical dendrites of upper motor neurons (UMNs) that become diseased from misfolded SOD1 toxicity. (A) Representative images of UMN apical dendrites in the motor cortex of WT‐UeGFP or (B) hSOD1^G93A^‐UeGFP mice treated with vehicle, (C) 20 mg/kg/day NU‐9, or (D) 100 mg/kg/day NU‐9. Boxed areas are enlarged to the right and additional examples are supplied. Scale bars: 50 µm (low mag), 10 µm (high mag inset); *n* ≥ 6 biological replicates. (E) Representative image of a healthy, intact, and (F) a diseased, disintegrating apical dendrite. Scale bars: 5 µm. (G) Average percentage of UMN apical dendrites with vacuoles per section in the motor cortex; mean, SEM, and individual data points shown for *n* ≥ 6 biological replicates. *p < .05, **p < .01, one‐way ANOVA followed by Tukey's post hoc multiple‐comparison test

Because the apical dendrite is the site of cortical integration and its stability is the key to proper UMN modulation, function, and health, we next investigated whether NU‐9 treatment would also improve the cytoarchitectural integrity of UMN apical dendrite and reduce the extent of its vacuolization and disintegration. WT‐UeGFP mice treated with vehicle have mostly healthy apical dendrites that extend toward the top layers, and only a small percentage had vacuoles (28% ± 12%; Figure 4A,E). WT‐UeGFP mice treated with 100 mg/kg/day of NU‐9 also have healthy apical dendrites with few vacuoles (16% ± 5%; adjusted *p*‐value = .9235). On the other hand, most of hSOD1^G93A^‐UeGFP UMNs treated with vehicle continued to have vacuolated and disintegrating apical dendrites (76% ± 11%; Figure 4B,F), and the difference between WT‐UeGFP controls was highly significant (adjusted *p*‐value = .0133; Figure 4G). However, NU‐9 treatment significantly improved the integrity of disintegrating apical dendrites in hSOD1^G93A^‐UeGFP mice in a dose‐dependent manner (Figure 4C,D). Upon 20 mg/kg/day treatment (Figure 4C), the percentage of UMNs with vacuolated apical dendrites was reduced to 44% ± 7%, and this is further reduced to 23% ± 10% when hSOD1^G93A^‐UeGFP mice are treated with 100 mg/kg/day of NU‐9 (Figure 4C,D,G; adjusted *p*‐value = .0019), which was comparable to the integrity of apical dendrites in the control WT‐UeGFP mice (adjusted *p*‐value = .9956).

### 3.6 NU‐9 treatment significantly improves UMN retention in the motor cortex of hSOD1^G93A^ mice

**FIGURE 5:**
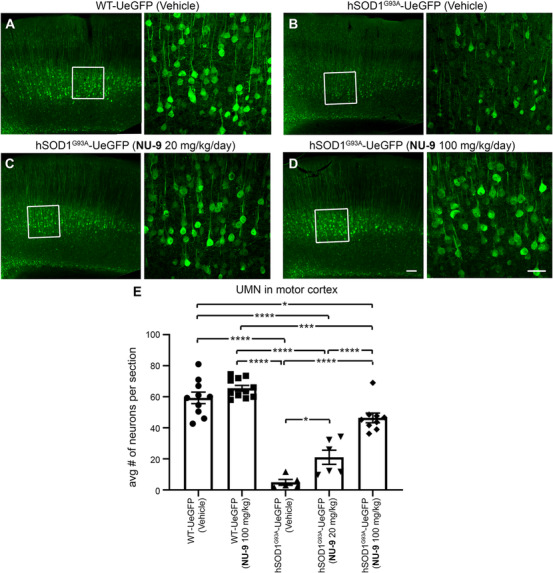
NU‐9 treatment reduces upper motor neuron (UMN) degeneration of UMNs diseased resulting from misfolded SOD1 toxicity *in vivo*. (A–D) Representative images of UMNs in the motor cortex of WT‐UeGFP or hSOD1^G93A^‐UeGFP mice treated with vehicle, 20 mg/kg/day NU‐9, or 100 mg/kg/day NU‐9. Scale bars: 50 µm; *n* ≥ 5 biological replicates. (E) Average number of UMNs per section in the motor cortex; mean, SEM, and individual data points shown for *n* ≥ 5 biological replicates. *p < .05, ***p < .001, ****p < .0001, One‐way ANOVA followed by Tukey's post hoc multiple‐comparison test

The timing and the extent of UMN loss in hSOD1^G93A^‐UeGFP mice^57^ is comparable to that of UMN loss in hSOD1^G93A^ mice.^60^ Vehicle treatment did not have an impact on UMN numbers in WT‐UeGFP mice (59 ± 3 UMNs; Figure 5A) or hSOD1^G93A^‐UeGFP mice (5 ± 2 UMNs; Figure 5B). UMN loss with respect to disease progression remained significant (adjusted *p*‐value < .0001) with vehicle treatment. NU‐9 treatment even at the 100 mg/kg/day dose did not have any adverse effects on UMN numbers in WT‐UeGFP mice (66 ± 1 UMNs; adjusted *p*‐value = .5368). However, when hSOD1^G93A^‐UeGFP mice were gavage treated daily with NU‐9, more UMN cell bodies were detected in the motor cortex (Figure 5C,D). There were 21 ± 5 UMN after 20 mg/kg/day of NU‐9 treatment (adjusted *p*‐value = .0446; Figure 5E), and upon treatment with a 100 mg/kg/day dose, there was a dramatic increase in the numbers of UMNs retained in the motor cortex (46 ± 3 UMN), which was highly significant when compared to vehicle‐treated mice (adjusted *p*‐value < .0001) and mice treated with 20 mg/kg/day (adjusted *p*‐value < .0001; Figure 5E). Importantly, the average number of UMN present in the motor cortex of hSOD1^G93A^‐UeGFP mice treated 60 days with 100 mg/kg/day of NU‐9 became almost comparable to that of UMN numbers present in healthy WT‐UeGFP mice (adjusted *p*‐value = .0306).

### 3.7 NU‐9 improves integrity of mitochondria and ER of UMNs with TDP‐43 pathology

**FIGURE 6:**
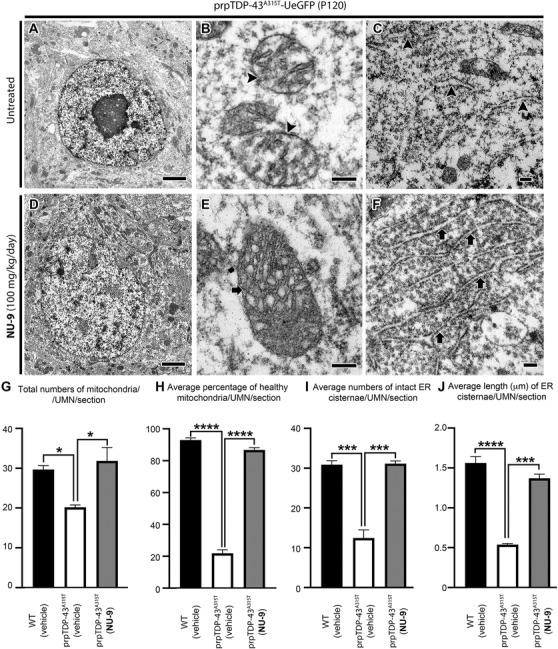
NU‐9 treatment improves ultrastructural integrity of both mitochondria and endoplasmic reticulum (ER) of upper motor neurons (UMNs) that become diseased due to TDP‐43 pathology. Mitochondrial and ER defects in the UMNs of prpTDP‐43^A315T^‐UeGFP mice were previously published. (A–C) Representative electron microscopic images of UMNs of untreated prpTDP‐43^A315T^‐UeGFP mice. (A) UMN soma, with few intact organelles. (B) Mitochondria lose integrity of their inner membrane (arrowheads), and (C) ER cisternae are disintegrated and broken (arrowheads). (D–F) Representative electron microscopic images of UMNs of prpTDP‐43^A315T^‐UeGFP mice treated with 100 mg/kg/day NU‐9. (D) An overall improvement in the cytoarchitecture is evidenced with proper nuclear membrane, presence of numerous healthy organelles, and lack of electron dense aggregates. (E) Mitochondria appear healthy with improved inner membrane and cristae (arrow) (F), and ER cisternae arranged in proper structure with ribosomes attached (arrows). Scale bars: A,D: 2 µm; B,C,E,F: 200 nm. (G) Quantification of the total number of mitochondria/UMN in prpTDP‐43^A315T^‐UeGFP mice with 100 mg/kg/day NU‐9 treatment; *p < .03. (H) Quantification of percentage of healthy mitochondria/UMN in prpTDP‐43^A315T^‐UeGFP mice with 100 mg/kg/day NU‐9 treatment; ****p < .0001. (I) Quantification of the number of ER cisternae/UMN in prpTDP‐43^A315T^‐UeGFP mice with 100 mg/kg/day NU‐9 treatment; ***p < .0003. (J) Quantification of average length of ER cisternae/UMN in prpTDP‐43^A315T^‐UeGFP mice with 100 mg/kg/day NU‐9 treatment; ****p < .0001. One‐way ANOVA followed by Tukey's post hoc multiple‐comparison test was used for statistical analyses

We recently discovered that cellular defects, which occurred in UMNs of Betz cells of ALS patients with TDP‐43 pathology, were fully recapitulated in the UMNs of TDP‐43^A135T^ mice.^34^ EM helped visualize and reveal intracellular defects that occur in UMNs (Figure 6A). Mitochondria were severely affected, their inner membrane was broken, and mitochondria disintegrated (Figure 6B arrowheads). Likewise, ER lost the integrity of their architecture; cisternae were broken (Figure 6C arrowheads), and pieces of ER with ribosomes attached were detected in the soma.

Since NU‐9 improved ultrastructural integrity of both mitochondria and ER of UMN diseased due to mSOD1 toxicity, and these were indeed the prominent defects detected in the UMNs with TDP‐43 pathology, we reasoned that a mechanism‐based treatment strategy would suggest NU‐9 to improve the mitochondrial and ER defects observed in UMNs that become diseased by TDP‐43 pathology as well, even though these are two distinct and different disease models. To visualize diseased UMNs and to assess their cellular response to compound treatment, TDP‐43^A315T^ mice^58^ were crossed with UCHL1‐eGFP mice^57^ to generate an UMN reporter disease model prpTDP‐43^A315T^‐UeGFP mice.^34^ There is no misfolded SOD1 detected in the UMN of prpTDP‐43^A315T^‐UeGFP mice (Figure S3), and there is no TDP‐43 pathology reported in SOD1 mouse models or patients with SOD1 mutations,^46^, ^47^, ^48^, ^49^, ^50^, ^51^ in fact mSOD1 toxicity and TDP‐43 pathology are accepted to be distinct causes of motor neuron degeneration. Therefore, UMN degeneration in TDP model cannot be explained by mSOD1 toxicity. We decided to take the leap and investigated whether NU‐9 treatment would also be effective with respect to TDP‐43 pathology (Figure 6A–C).

prpTDP‐43^A315T^‐UeGFP mice (*n* = 4) were treated with a 100 mg/kg/day dose of NU‐9, and sex‐ and age‐matched untreated prpTDP‐43^A315T^‐UeGFP mice (*n* = 3) were used as the negative control. As both hSOD1^G93A^ and prpTDP‐43^A315T^ mice were mated with the same WT‐UeGFP mouse colony to generate hSOD1^G93A^‐UeGFP and prpTDP‐43^A315T^‐UeGFP mice, same WT‐UeGFP cohort was used as healthy control for both groups.

NU‐9 treatment resulted in profound improvements in both mitochondria and ER of UMNs that become diseased as a result of TDP‐43 pathology (Figure 6D–F). Mitochondria, especially the inner membranes of mitochondria, became intact (Figure 6E arrow), and there were no signs of mitoautophagy^38^ or mitophagy. The ER retained its structure with ribosomes attached, and there was no enlargement or disintegration of cisternae (Figure 6F arrows). Quantitative analysis confirmed significant increase in the numbers of total mitochondria per UMN per section after 100 mg/kg/day NU‐9 treatment (32 ± 3 mitochondria/UMN/section), when compared to mitochondria in diseased UMN (prpTDP‐43^A315T^‐UeGFP: 20 ± 1 mitochondria/UMN/section; *p* < .033; Figure 6G). Interestingly, the number of mitochondria after NU‐9 treatment in prpTDP‐43^A315T^‐UeGFP mice became comparable to that of WT mice (30 ± 1 mitochondria/UMN/section; adjusted *p*‐value = .76). The average percentage of healthy mitochondria also significantly increased with NU‐9 treatment (87% ± 1% mitochondria/UMN/section) when compared to diseased UMNs (prpTDP‐43^A315T^‐UeGFP: 22% ± 2% mitochondria/UMN/section; adjusted *p*‐value = .0001; Figure 6H) and were comparable to the average percentage of healthy mitochondria in WT‐UeGFP mice (93 ± 1 mitochondria/UMN/section).

NU‐9 treatment also increased the average number of intact ER cisternae in UMNs that become diseased as a result of TDP‐43 pathology (WT‐UeGFP: 31 ± 1 ER cisternae/UMN/section; prpTDP‐43^A315T^‐UeGFP: 12 ± 2 ER cisternae/UMN/section; prpTDP‐43^A315T^‐UeGFP [100 mg/kg/day NU‐9]: 36 ± 2 ER cisternae/UMN/section, *n* = 4 mice, adjusted *p*‐value = .0002; Figure 6I). The average length of ER cisternae became also significantly longer in the UMN treated with NU‐9 (WT‐UeGFP: 1.56 ± 0.07 µm; prpTDP‐43^A315T^‐UeGFP: 0.53% ± 0.01%; adjusted *p*‐value = .0001; prpTDP‐43^A315T^‐UeGFP treated with NU‐9: 1.37% ± 0.05%; adjusted *p*‐value = .0001; Figure 6J).

### 3.8 NU‐9 treatment eliminates degeneration of UMNs with TDP‐43 pathology in vivo

We previously reported problems with mitochondria and ER of UMNs with TDP‐43 pathology,^34^ and because NU‐9 treatment enhanced the integrity of mitochondria and the ER of diseased neurons at the ultrastructural level (Figure 6), we next investigated whether NU‐9 treatment would also support cellular integrity and survival of UMN with TDP‐43 pathology*in vivo*.

**FIGURE 7:**
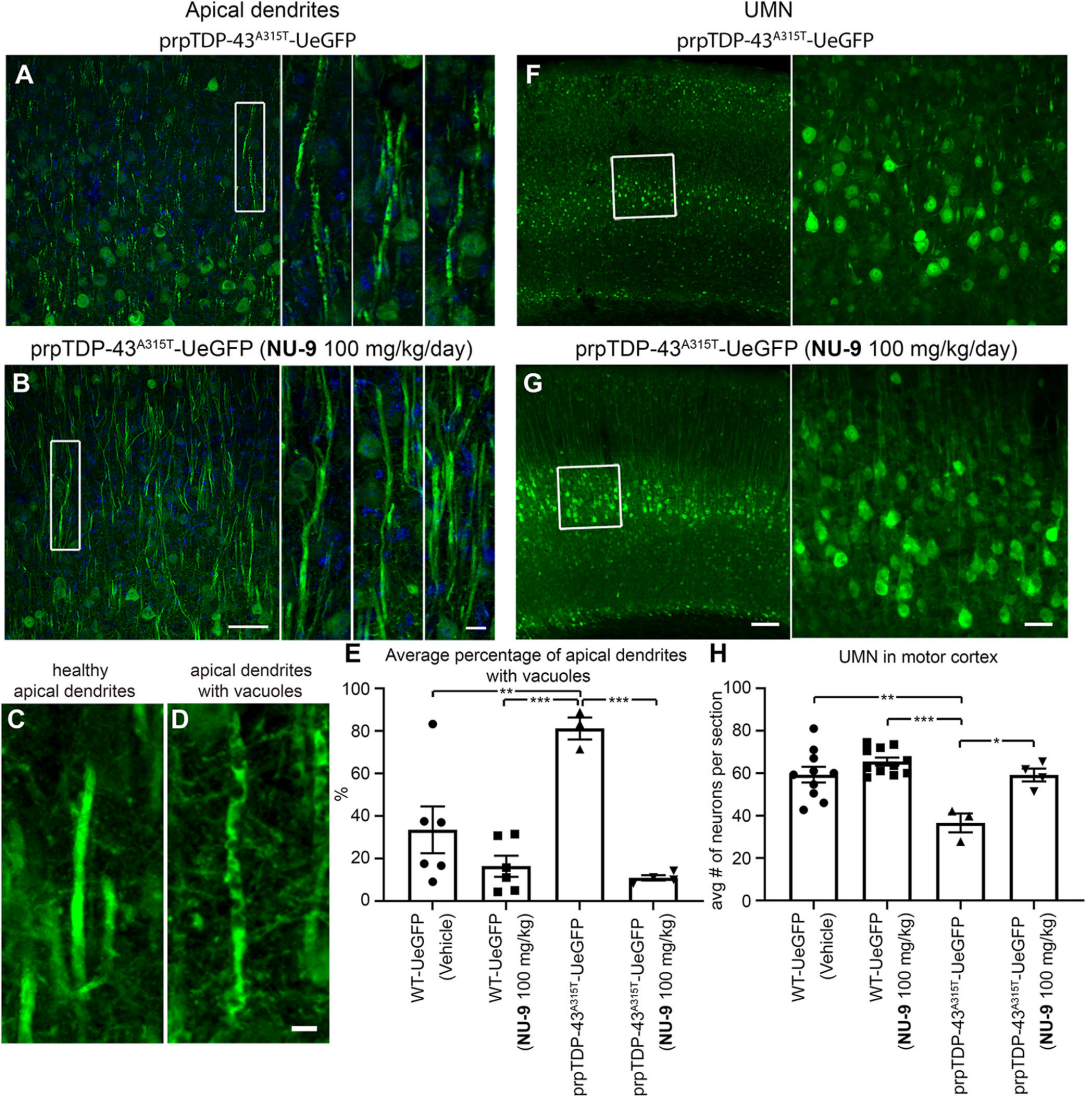
NU‐9 treatment improves cytoarchitectural integrity of disintegrating apical dendrites and eliminates progressive degeneration of upper motor neurons (UMNs) that become diseased due to TDP‐43 pathology. (A) Representative images of UMN apical dendrites in the motor cortex of untreated prpTDP‐43^A315T^‐UeGFP mice and (B) prpTDP‐43^A315T^‐UeGFP mice treated with 100 mg/kg/day of NU‐9. Boxed area is enlarged to the right with additional representative examples. Scale bars: 50 µm (low mag), 10 µm (high mag inset); *n* ≥ 3 biological replicates. (C) Representative image of a healthy, intact, and (D) a diseased, disintegrating apical dendrite. Scale bars: 5 µm. (E) Average percentage of apical dendrites with vacuoles per section area in the motor cortex; mean, SEM, and individual data points shown for *n* ≥ 3 biological replicates. **p < .01, ***p < .001, One‐way ANOVA followed by Tukey's post hoc multiple‐comparison test. (F) Representative images of UMNs in the motor cortex of untreated prpTDP‐43^A315T^‐UeGFP mice and (G) prpTDP‐43^A315T^ treated with 100 mg/kg/day NU‐9. Scale bars: 50 µm; *n* ≥ 3 biological replicates. (H) Average number of UMNs per section area in the motor cortex; mean, SEM, and individual data points shown for *n* ≥ 3 biological replicates. *p < .05, **p < .01, ***p < .001, One‐way ANOVA followed by Tukey's post hoc multiple‐comparison test

Health and integrity of apical dendrites in prpTDP‐43^A315T^‐UeGFP mice displayed profound improvement with NU‐9 treatment as the percentage of UMNs with vacuolated primary apical dendrites was significantly reduced (untreated: 81% ± 5%; NU‐9 [100 mg/kg/day]: 11% ± 1%; adjusted *p*‐value = .0005; Figure 7A–E). Most interestingly, the average number of UMNs in the motor cortex of prpTDP‐43^A315T^‐UeGFP mice treated with NU‐9 experienced a profound increase compared to that of diseased untreated prpTDP‐43^A315T^–UeGFP mice (untreated: 37 ± 4 UMNs; NU‐9 [100 mg/kg/day]: 59 ± 3 UMNs; adjusted *p*‐value = .0121; Figure 7F–H). Same WT‐UeGFP cohort was used as healthy control for both hSOD1^G93A^‐UeGFP and prpTDP‐43^A315T^‐UeGFP mice, as previously mentioned. The UMN numbers with NU‐9 treatment were comparable and almost identical to those of the healthy control mice treated with vehicle (WT‐UeGFP mice 59 ± 4 UMNs; adjusted *p*‐value > .9999), revealing the ability of NU‐9 to eliminate the ongoing degeneration of UMNs that become diseased due to TDP‐43 pathology.

### 3.9 Effect of NU‐9 on lower motor neurons (LMNs)

**FIGURE 8:**
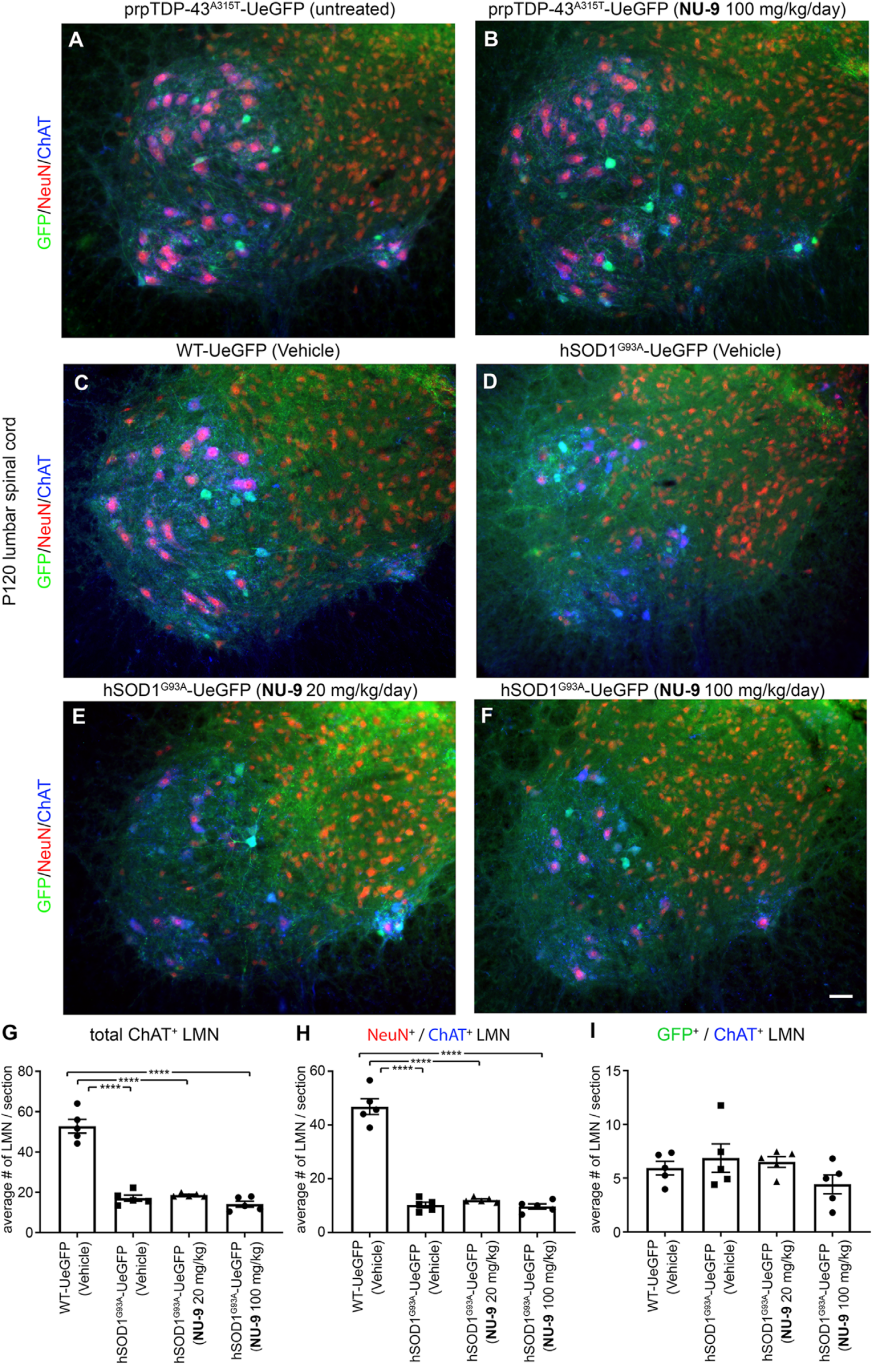
NU‐9 treatment does not improve lower motor neuron (LMN) degeneration resulting from misfolded SOD1 toxicity *in vivo*. (A and B) Representative images of LMNs in the lumbar spinal cord of untreated prpTDP‐43^A315T^‐UeGFP mice or treated with 100 mg/kg/day NU‐9. Scale bar: 50 µm; *n* ≥ 3 biological replicates. (C–F) Representative images of LMNs in the lumbar spinal cord of WT‐UeGFP or hSOD1^G93A^‐UeGFP mice treated with vehicle, 20 mg/kg/day NU‐9, or 100 mg/kg/day NU‐9. Scale bar: 50 µm; *n* = 5 biological replicates. (G) Average number of ChAT^+^ LMNs per section in the lumbar spinal cord; mean, SEM, and individual data points shown for *n* = 5 biological replicates. ****p < .0001, One‐way ANOVA followed by Tukey's post hoc multiple‐comparison test. (H) Average number of NeuN^+^/ChAT^+^ LMNs (vulnerable to degeneration) per section in the lumbar spinal cord; mean, SEM, and individual data points shown for *n* = 5 biological replicates. ****p < .0001, One‐way ANOVA followed by Tukey's post hoc multiple‐comparison test. (I) Average number of GFP^+^/ChAT^+^ LMNs (resistant to degeneration) per section in the lumbar spinal cord; mean, SEM, and individual data points shown for *n* = 5 biological replicates

In an effort to determine whether NU‐9 treatment also improves the health and survival of lower motor neurons (LMNs), we investigated the lumbar spinal cords of both hSOD1^G93A^‐UeGFP and prpTDP‐43^A315T^‐UeGFP mice (Figure 8). As indicated by previous reports,^58^, ^66^ there was no prominent LMN loss in the spinal cords of prpTDP‐43^A315T^‐UeGFP mice, even at P120 (Figure 8A,B), and thus investigation of NU‐9 treatment on the survival of LMNs was not possible. However, as extensively reported in the field,^59^, ^67^, ^68^, ^69^ there was a dramatic reduction in the numbers of LMNs in hSOD1^G93A^‐UeGFP mice (17.1 ± 1.3 LMN) when compared to the healthy controls (52.8 ± 3 LMN; adjusted *p*‐value < .0001; Figure 8C,D). We quantitatively assessed the changes in the numbers of LMNs in the lumbar spinal cord of mice that are treated with vehicle, NU‐9 (20 or 100 mg/kg/day), and control healthy mice (Figure 8E,F). NU‐9 treatment, regardless of dose, was not sufficient to eliminate ongoing LMN degeneration in hSOD1^G93A^‐UeGFP mice. There was no difference in LMN numbers between vehicle and treated (NU‐9 [20 mg/kg/day]: 18.6 ± 0.4 LMN; vs. vehicle adjusted *p*‐value = .9526; NU‐9 [100 mg/kg/day]: 14.1 ± 1.3 LMN; vs. vehicle adjusted *p*‐value = .7255) and untreated mice at P120 (Figure 8E,F; Table S5).

As the UCHL1‐eGFP reporter selectively labels small diameter alpha and gamma motor neurons that are not vulnerable to degeneration in ALS,^57^, ^68^, ^69^, ^70^, ^71^ we investigated both the NeuN^+^/ChAT^+^ LMN subpopulation vulnerable to degeneration and GFP^+^/ChAT^+^ LMN subpopulation resistant to degeneration. There was a dramatic reduction in the numbers of NeuN^+^/ChAT^+^ LMNs in the diseased mice (10.3 ± 0.9 LMN) when compared to the healthy controls (46.8 ± 2.6 LMN; adjusted *p*‐value < .0001; Figure 8F). However, NU‐9 treatment, regardless of dose, was not sufficient to eliminate LMN degeneration in hSOD1^G93A^‐UeGFP mice; there was no statistical difference in NeuN^+^/ChAT^+^ LMN numbers between treated (NU‐9 [20 mg/kg/day]: 12.1 ± 0.4 LMN; vs. vehicle: 10.3 ± 0.9; adjusted *p*‐value = .8524; NU‐9 [100 mg/kg/day]: 9.7 ± 0.9 LMN; vs. vehicle: 10.3 ± 0.9; adjusted *p*‐value = .995; Figure 8H) and untreated mice at P120. The numbers of GFP^+^ and ChAT^+^ LMN were comparable, regardless of genotype or treatment (Figure 8I, Table S5), revealing neuroprotective effects of NU‐9 to be selective for UMNs.

### 3.10 NU‐9 treatment improves upper motor neuron function

**FIGURE 9:**
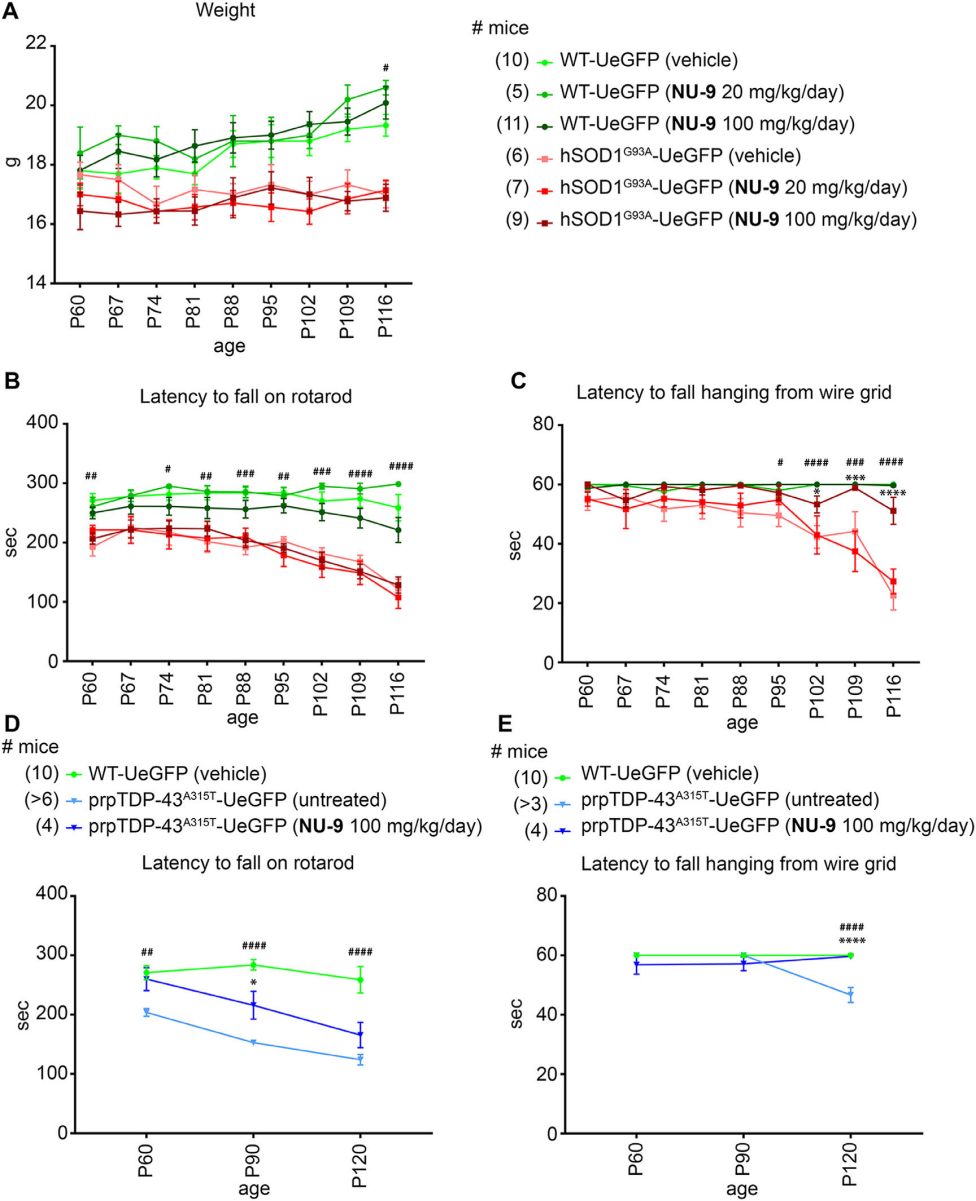
Behavioral data of WT‐UeGFP, hSOD1^G93A^‐UeGFP, and prpTDP‐43^A315T^‐UeGFP mice with or without NU‐9 treatment. WT‐UeGFP and hSOD1^G93A^‐UeGFP mice were tested every 7 days between postnatal day (P)60 and P120; prpTDP‐43^A315T^‐UeGFP mice were tested at P60, P90, and P120. (A) Weight of the mice in grams. Mean and SEM shown for *n *≥ 5 mice per group. WT‐UeGFP (vehicle) versus hSOD1^G93A^‐UeGFP (vehicle); ^#^ *p* < .05, two‐way ANOVA with Tukey's multiple‐comparison test. (B) Latency to fall on accelerating rotarod in seconds. Mean and SEM shown for *n *≥ 5 mice per group. WT‐UeGFP (vehicle) versus hSOD1^G93A^‐UeGFP (vehicle). ^#^ *p* < .05, ^##^ *p* < .01, ^###^ *p* < .001, ^####^ *p* < .0001, Two‐way ANOVA with Tukey's multiple‐comparison test. (C) Latency to fall hanging upside down from a wire grid in seconds. Mean and SEM shown for *n *≥ 5 mice per group. WT‐UeGFP (vehicle) versus hSOD1^G93A^‐UeGFP (vehicle). ^#^ *p* < .05, ^###^ *p* < .001, ^####^ *p* < .0001; hSOD1^G93A^‐UeGFP (vehicle) versus hSOD1^G93A^‐UeGFP (NU‐9 100 mg/kg/day). *p < .05, ***p < .001, ****p < .0001, Two‐way ANOVA with Tukey's multiple‐comparison test. (D) Latency to fall on accelerating rotarod in seconds. Mean and SEM shown for *n *≥ 4 mice per group. WT‐UeGFP (vehicle) versus prpTDP‐43^A315T^‐UeGFP (vehicle). ^##^ *p* < .01, ^####^ *p* < .0001, Mixed‐effects analysis with Tukey's multiple‐comparison test. (E) Latency to fall hanging upside down from a wire grid in seconds. Mean and SEM shown for *n *≥ 3 mice per group. WT‐UeGFP (vehicle) versus prpTDP‐43^A315T^‐UeGFP (vehicle), ^####^ *p* < .0001; prpTDP‐43^A315T^‐UeGFP (untreated) versus prpTDP‐43^A315T^‐UeGFP (NU‐9 100 mg/kg/day), ****p < .0001, mixed‐effects analysis with Tukey's multiple‐comparison test

Even though most behavioral assays fail to properly assess UMN health and connectivity, the hanging wire test is reported to be more specific to UMN integration, as evidenced by Fezf2 −/− mice that are born without UMN and CST axons, which perform very poorly in this test.^72^ Therefore, we performed both the well‐studied rotarod^73^, ^74^ and the hanging wire test, which reveals the ability of the mouse to use its fingers and digits to hold on to the wire (Figure 9).

There was a distinction between healthy WT‐UeGFP and diseased hSOD1^G93A^‐UeGFP mice on the hanging wire test, as hSOD1^G93A^‐UeGFP mice failed to grab and hold on to the inverted wire as disease progressed. The difference became significant by P95 and continued to be significant throughout (P95: WT‐UeGFP: 60 s; hSOD1^G93A^‐UeGFP: 49.5 ± 3.7 s, adjusted *p*‐value = .0328; Figure 9C). Untreated hSOD1^G93A^‐UeGFP mice were not able to stay on the hanging wire and their performance continued to decline with age (Figure 9C, Table S3). On the contrary, hSOD1^G93A^‐UeGFP mice treated with a 100 mg/kg/day dose of NU‐9 performed significantly better than hSOD1^G93A^‐UeGFP mice treated with vehicle by P102 (hSOD1^G93A^‐UeGFP [vehicle]: 42.3 ± 3.8 s; hSOD1^G93A^‐UeGFP [100 mg/kg/day NU‐9]: 53.3 ± 2.9 s; adjusted *p*‐value = .0254), and this performance was comparable to that of healthy mice at that age (WT‐UeGFP [vehicle]: 60 s; adjusted *p*‐value = .2529). Unlike hanging wire test, NU‐9 treatment did not result in significant improvement in rotarod performance, regardless of dose (Figure 9B, Table S3).

prpTDP‐43^A315T^ mice performed worse than WT littermates on rotarod and hanging wire tests.^34^, ^75^, ^76^ However, when treated with a 100 mg/kg/day dose of NU‐9, they performed significantly better on the hanging wire test, so much so that they became comparable to healthy WT mice at P120 (untreated prpTDP‐43^A315T^‐UeGFP: 46 ± 7 s; prpTDP‐43^A315T^ treated with NU‐9: 59.5 ± 0.3 s; adjusted *p*‐value < .0001; WT‐UeGFP [vehicle]: 60 ± 0 s; vs. untreated prpTDP‐43^A315T^‐UeGFP adjusted *p*‐value < .0001 WT vehicle vs. prpTDP‐43^A315T^‐UeGFP treated with NU‐9 [100 mg/kg/day] adjusted *p*‐value = .9688; Figure 9E, Table S3).

Different from hSOD1^G93A^ mouse model, even the rotarod test revealed significant improvement in TDP‐43 model only after 30 days of NU‐9 treatment (untreated prpTDP‐43^A315T^‐UeGFP: 153.1 ± 4.8 s; prpTDP‐43^A315T^‐UeGFP treated with NU‐9 [100 mg/kg/day]: 232.6 ± 18.1 s; adjusted *p*‐value = .0482; WT‐UeGFP [vehicle]: 283.9 ± 8.9 s; vs. untreated prpTDP‐43^A315T^‐UeGFP; adjusted *p*‐value < .0001 WT vehicle vs. prpTDP‐43^A315T^‐UeGFP treated with NU‐9 [100 mg/kg/day] adjusted *p*‐value = .0482; Figure 9D; please refer to Table S3 for all values and statistical analysis at each time point).

## 4 DISCUSSION

The cellular events that give rise to selective neuronal vulnerability leading to neurodegenerative diseases are now better understood than a decade ago,^77^, ^78^, ^79^, ^80^, ^81^, ^82^, ^83^, ^84^ and many more compounds are generated and characterized with drug‐like properties.^85^, ^86^, ^87^, ^88^ Yet, there has been no effective cure for any of the motor neuron diseases, especially for the diseases of the UMNs. One of the major limitations has been the lack of proper tools and drug discovery platforms that would utilize UMN response as the readout. In their absence, preclinical assays rely heavily on extension of mouse lifespan as outcome measure.^14^, ^89^, ^90^, ^91^, ^92^, ^93^ However, the lack of translation from mouse models to humans has resulted in numerous failed clinical trials and compounded frustrations. The need to develop better preclinical assays that provide information about the survival needs of vulnerable and degenerating neurons in patients has become evident.^40^, ^41^, ^94^

Even though UMNs are a critical component of motor neuron circuitry, the idea that their degeneration is secondary to LMN loss and is a byproduct of an ongoing degeneration, previously diminished their importance as a potential cellular target for therapeutic interventions.^67^, ^95^, ^96^ However, mounting experimental data now reveal that the cellular pathology of UMNs becomes evident much earlier than symptom onset.^32^, ^36^, ^37^, ^57^ Spine loss and apical dendrite degeneration occurs prior to neuronal loss,^97^, ^98^, ^99^, ^100^ and cortical hyperexcitation is even used as an early detection marker of ALS.^35^, ^101^, ^102^, ^103^

In a rat model of mSOD1, the reduction of G93A mutation levels only in the motor cortex improved the health and integrity of global motor neuron circuitry,^104^ further supporting the idea that UMNs are feasible targets. Recent studies also confirmed the importance and relevance of UMNs in ALS pathology. When UMNs were ablated from the SOD1 mouse models by crossing with the fezf2 null, the results revealed that UMNs indeed play an important role for initiating and modulating disease pathology and that their degeneration is not by mere consequence.^72^, ^105^ In addition, floxed hSOD1^G37R^ mice recapitulate UMN loss. When mutant hSOD1^G37R^ is removed from UMN of floxed mice by crossing them with the CrymCreER^T2^ mice, UMN loss is prevented suggesting that UMN degeneration relies on cell autonomous mechanism.^106^ These recent findings establish UMNs as important contributors to disease pathology in ALS and further suggest that their survival needs to be considered for building effective treatment strategies.

The second reason that has diminished interest in UMNs has been the assumption that treatments that benefit LMNs should also be beneficial to UMNs, and that since they are both motor neuron populations, they do not need to be investigated separately. This could be the reason why none of the compounds that have been in clinical trials for ALS have ever been tested for their ability to improve UMN health. It has been assumed that if a compound improves the health and integrity of LMNs, it should also improve the health of UMNs, and therefore no special or additional emphasis has been given to the UMNs. The assumption that these two neuron populations are similar is unfounded because they are born from different progenitors, and their differentiation, maturation, target recognition, and integration to circuitry occur at different time points, at different sites, and via different molecular mechanisms.^107^ Their gene expression profiles and neuronal identities also are very different. Thus, it is not reasonable to think that they should respond similarly to treatment. In fact, their requirements for survival could indeed be very different.^108^, ^109^, ^110^

By focusing our attention on the needs of diseased neurons, and building effective treatment strategies that take their survival requirements into account, we could set the stage for treatments that are translational.^41^, ^94^ We have developed a novel platform in which the responses of UMNs to compound treatment can be readily assessed at a cellular level with precision and clarity that was not previously possible. Our study revealed that NU‐9 treatment improved the ultrastructural integrity of mitochondria and the ER, both of which are exceptionally important organelles for motor neuron health.^34^, ^38^

Mitochondria are responsible for the generation of ATP.^111^ They also play a key role in the initiation of innate immunity.^112^ Therefore, the health and integrity of mitochondria are crucial for motor neurons that have high levels of energy demand and must control neuroimmune reactions for improved health.^113^, ^114^, ^115^ Mitochondrial problems occur very early and selectively in UMNs, which develop the disease because of mSOD1 toxicity, lack of Alsin function, and Profilin mutations.^34^, ^37^, ^38^, ^57^, ^110^, ^116^, ^117^ Misfolded SOD1 selectively binds to mitochondria and affects their shape and function.^118^, ^119^, ^120^, ^121^, ^122^, ^123^, ^124^, ^125^ TDP‐43 also binds to mitochondria, and inhibiting TDP‐43 binding to mitochondria improves motor neuron function.^126^, ^127^, ^128^ Therefore, reducing the levels of misfolded SOD1 and TDP‐43 pathology potentially improves mitochondrial function. Likewise, improving mitochondrial function also reduces levels of misfolded SOD1 in motor neurons.^125^ Therefore, further studies are required to reveal the details of cause and effect.^129^

The ER is the site of protein production and initial folding, where defects result in ER stress, one of the converging pathologies shared among many different neurodegenerative diseases.^130^ We previously reported that diseased UMNs are especially prone to ER stress, and increased ER stress contributes to their early vulnerability, while other cortical neurons remain healthy.^36^ Electron microscopy studies reveal the presence of ER stress as a result of the enlargement of lumen, followed by disintegration of the ER.^34^ The ability of NU‐9 to improve the integrity of both mitochondria and the ER is exceptionally significant, because, even though the underlying causes of the disease are heterogeneous, many of the pathways converge on the health and function of mitochondria and the ER.^131^, ^132^, ^133^ Disruption of intracellular membrane organelles, such as the Golgi apparatus, has been suggested as a possible cause for ALS^134^ and is proposed to be upstream of the ER dysfunction.^135^ Maintenance of the ultrastructural integrity of these two key organelles, especially in the neurons that display primary vulnerability, would enable them to perform their much required function. This may explain why NU‐9 treatment improves UMN cytoarchitecture and eliminates their progressive degeneration in both hSOD1^G93A^ and TDP‐43^A315T^ mice.

The underlying pathologies of the UMNs in well‐characterized mouse models and UMNs in patients with motor neuron diseases are almost identical at the cellular level. For example, the apical dendrite degeneration observed in UMNs was also recapitulated in the Betz cells of fALS, sALS, and ALS with FTLD patients.^8^ The apical dendrite is exceptionally important for the function of UMNs. We previously identified apical dendrite defects in UMNs that become diseased by mSOD1 toxicity,^37^, ^57^ lack of Alsin function,^116^ Profilin defects,^117^ and TDP‐43 pathology,^32^ and these cellular defects were fully recapitulated in the Betz cells—the UMNs in humans—of a broad spectrum of ALS patients, including sALS, fALS, as well as ALS/FTLD.^8^ UMNs of patients had vacuolated apical dendrites, which display massive disintegration.^8^, ^32^, ^36^, ^37^ This is the site where other cortical neurons connect and communicate with the UMNs.^8^, ^35^, ^136^, ^137^ When apical dendrite disintegrates, it is not possible for UMNs to be properly modulated and thus they fail to convey cerebral cortex's signal to the spinal cord targets. We and others find that UMN apical dendrite degeneration is an early event in ALS and contributes to the dysregulation of cortical hyperexcitation and hypoexcitation even prior to symptom onset.^35^ Therefore, being able to reverse the ongoing apical dendrite degeneration would have significant outcomes for neuronal health and connectivity. We find that NU‐9 treatment enhances the cytoarchitectural stability of apical dendrite, such that they become comparable to WT healthy controls. These findings would have important implications for the health and connectivity of UMNs. One of the outcomes of improved neuronal connectivity *in vivo* is improved motor function. Even though detection of UMN involvement in motor function of mice is challenging, recent evidence suggests a unique utility for the hanging wire test to interrogate UMN involvement. We find that NU‐9 treatment improves the ability of both prpTDP‐43^A315T^ and hSOD1^G93A^ mice to do better in the hanging wire test, further suggesting that the observation of a cellular function has a direct consequence in motor behavior.

NU‐9 treatment not only improves the health of UMNs that become diseased as a result of mSOD1 toxicity but also because of TDP‐43 pathology, which represent two important, yet independent, causes of motor neuron degeneration. Mutations in the *SOD1* gene were identified in ALS patients,^138^ and the disease mouse models were based on the mutations detected in patients^59^; these models mimicked many aspects of human pathology, including progressive UMN loss.^57^, ^110^ It is important to note that misfolded SOD1 inclusions are also detected in patients with mutations in C9orf72 and other ALS/FTLD associated genes.^139^, ^140^ In addition, ER stress leads to accumulation of even the WT SOD1 aggregates.^141^Misfolded WT SOD1 thus can propagate in a prion‐like fashion and seed cytotoxic misfolding of WT SOD1.^142^, ^143^ Therefore, it is important to identify compounds that can reduce misfolded SOD1‐mediated toxicity.

TDP‐43 pathology develops regardless of a mutation in the TARDP gene,^144^, ^145^ and it is observed in the brains of about 95% of ALS patients.^146^ Most patients with SOD1 mutations do not display TDP‐43 pathology in their brains,^46^, ^50^ and TDP‐43 accumulations are not detected,^46^, ^47^, ^48^, ^50^ albeit interactions between mSOD1 and TDP‐43 have been suggested.^147^, ^148^, ^149^ In SOD1 mouse models with G93A, G37R or G85R mutations, there is no mislocalization of TDP‐43 to the cytoplasm in motor neurons of mutant SOD1 transgenic mice, nor association of TDP‐43 with ubiquitinated inclusions.^50^ In addition, abnormally phosphorylated or truncated TDP‐43 species were not detected in fractionated ALS mouse spinal cord or brain.^51^ DNA strand breaks and TDP‐43 mislocalization are absent in the murine hSOD1^G93A^ model of ALS both *in vivo* and *in vitro*.^49^ Therefore, identification of a compound that improves the health of UMNs that become diseased by these two prominent and distinct causes is rather significant. It also suggests that NU‐9 would have an impact in a broad spectrum of patients, including patients with ALS, HSP, PLS, and ALS/FTLD.

## 5 CONCLUSIONS

Today, drug discovery efforts are more focused on reversing the disease‐causing cellular mechanisms and improving the health of neurons that display selective and progressive degeneration. However, numerous challenges still exist, especially for the diseases of the UMNs. For example, the underlying causes of neuronal vulnerability are not well defined, and there has been no preclinical assay that assesses cellular responses of UMNs to compound treatment. None of the compounds that are in clinical trials for motor neuron diseases have ever been tested on diseased UMNs. Here, we first find that the mitochondrial defects and problems with ER are observed both in the UMNs of ALS patients and in the UMNs of mouse models that are developed to mimic patients with mSOD1 toxicity and TDP‐43 pathology. There is translation at a cellular level, and even though the UMNs are in different species, the underlying causes of UMN vulnerability are the same. Mitochondrial defects and problems with the ER, therefore, offer a target for intervention. We find that NU‐9, a compound that was previously characterized to reduce mSOD1 aggregates in cell lines and a compound that crosses the blood brain barrier with favorable pharmacokinetic properties, has the unique ability to improve the structure and the integrity of both mitochondria and ER. This unique ability results in enhancing the cytoarchitectural integrity of degenerating UMNs and, most importantly, stopping the progressive degeneration of UMNs that become diseased as a result of mSOD1 toxicity and TDP‐43 pathology, two independent and overarching causes of neurodegeneration. Our findings mark the identification of the first compound that improves the health of diseased UMNs, and lay the foundation for future mechanism‐focused and cell‐based drug discovery studies.

## CONFLICT OF INTEREST

The authors declare that there is no conflict of interest.

## AUTHOR CONTRIBUTIONS

Barış Genç, Mukesh Gautam, Öge Gözütok, Ina Dervishi, Santana Sanchez, Nuran Koçak, Edward Xie, and P. Hande Özdinler performed experiments. Gashaw M. Goshu generated the compound used in the study. Richard B. Silverman and P. Hande Özdinler analyzed the data and wrote the manuscript with other authors.

## Supporting information


**Figure S1** UMN were identified based on Ctip2 immunopositive nuclei for EM analysis
**Figure S2** Misfolded SOD1 accumulates in UMNs of hSOD1^G93A^‐UeGFP mice in layer 5 of motor cortex
**Figure S3** Misfolded SOD1 does not accumulate in UMNs of prpTDP‐43^A315T^‐UeGFP mice in layer 5 of motor cortex
**Table S1** Information about the postmortem brain samples utilized in this study
**Table S2** Number of mice included in the in vivo studies

**Table S3** Number of mice used for behavior analysis, average and SEM values for each group, and statistical analysis at each time point
**Table S4** Number of mice, total number of UMN, total number of mitochondria, and total number of ER cisternae used for electron microscopy analysis

**Table S5** Statistics for bar graphs shown in figures

## Data Availability

Data are not in archive. NU‐9 will be made available to other researchers through an MTA.
