# Supplementary material for: Improving mitochondria and ER stability helps eliminate upper motor neuron degeneration that occurs due to mSOD1 toxicity and TDP‐43 pathology
Source: Clin Transl Med. 2021 Feb 22;11(2):e336. doi: 10.1002/ctm2.336 (PMC7898037; doi:10.1002/ctm2.336)
Supplement: Supplementary file 1 — Figure S1 UMN were identified based on Ctip2 immunopositive nuclei for EM analysis Figure S2 Misfolded SOD1 accumulates in UMNs of hSOD1G93A‐UeGFP mice in layer 5 of motor cortex Figure S3 Misfolded SOD1 does not accumulate in UMNs of prpTDP‐43A315T‐UeGFP mice in layer 5 of motor cortex Table S1 Information about the postmortem brain samples utilized in this study Table S2 Number of mice included in the in vivo studies [file CTM2-11-e336-s001.pdf]

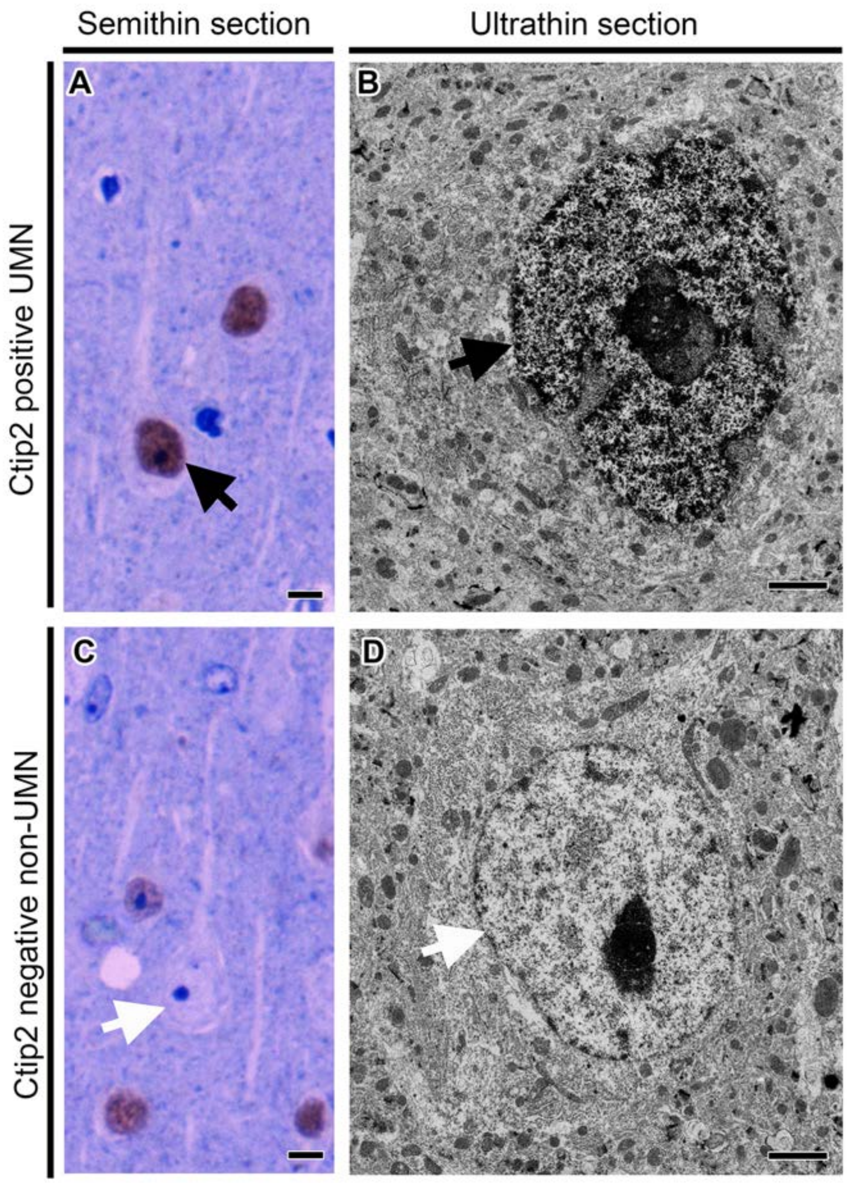

Figure S1. UMN were identified based on Ctip2 immunopositive nuclei for EM analysis. (**A**) Representative image of an UMN with Ctip2 positive nucleus (black arrow) as seen in semithin section. (**B**) Representative image of an UMN with electron dense DAB in its nucleus (black arrow) as seen in ultrathin section. (**C-D**) Representative images of a non-UMN cortical neuron with Ctip2 negative nuclei (white arrows) in semithin (**C**) and ultrathin (**D**) sections. Scale bars: **A**, **C** = 5 μm, **B**, **D** = 2 μm.

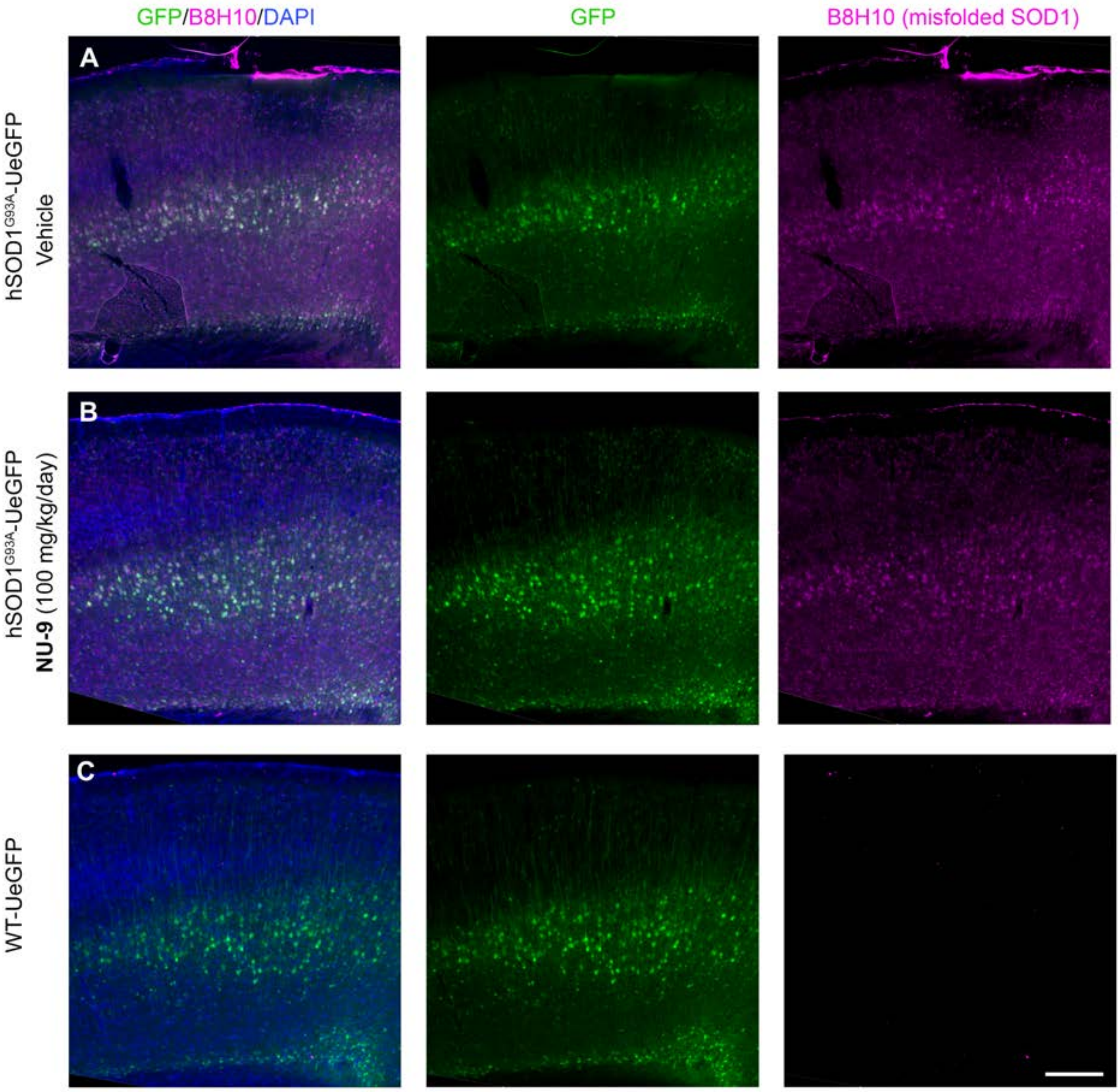

Figure S2. Misfolded SOD1 accumulates in UMNs of hSOD1^{G93A}-UeGFP mice in layer 5 of motor cortex. (**A**) Representative merged (first panel) images of UMNs (second panel) and B8H10 antibody staining (third panel) that recognizes misfolded SOD1 protein in the motor cortex of hSOD1^{G93A}-UeGFP mice treated with vehicle or (**B**) 100 mg/kg/day **NU-9**. (**C**) There is no misfolded SOD1 protein detected with B8H10 antibody in WT-UeGFP. Scale bars, 250 µm; n ≥ 3 biological replicates.

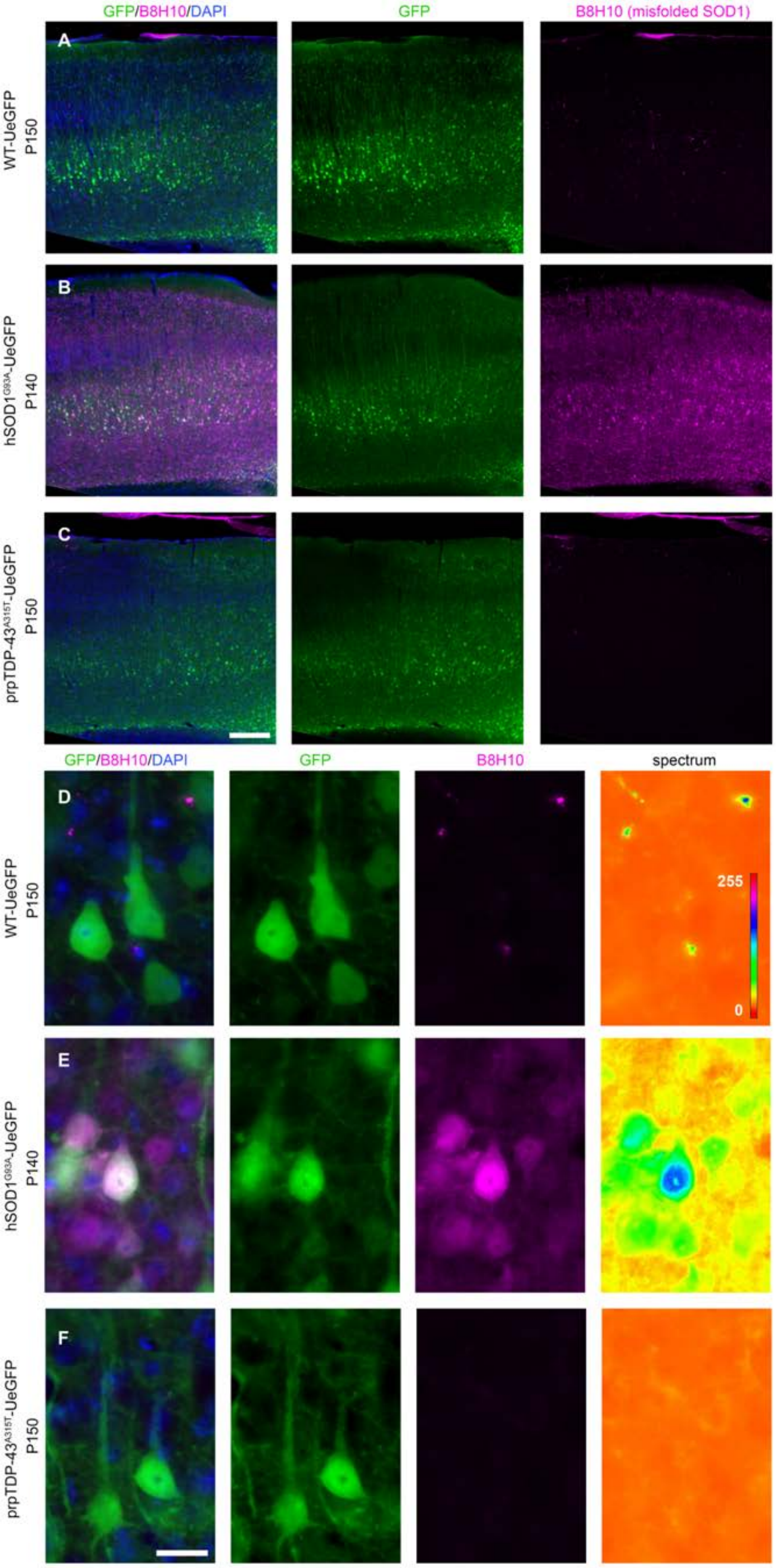
GFP/B8H10/DAPI
GFP
B8H10 (misfolded SOD1)
A
B
C
WT-UeGFP P150
hSOD1G93A-UeGFP P140
prpTDP-43A315T-UeGFP P150
GFP/B8H10/DAPI
GFP
B8H10
spectrum
D
E
F
255
0

Figure S3. Misfolded SOD1 accumulates in UMNs of hSOD1^{G93A}-UeGFP mice in layer 5 of motor cortex, but not prpTDP-43^{A315T} mice. (**A**) Representative merged (first panel) images of UMNs (second panel) and B8H10 antibody staining (third panel) that recognizes misfolded SOD1 protein in the motor cortex of untreated WT-UeGFP or (**B**) untreated end-stage hSOD1^{G93A}-UeGFP mice or (**C**) untreated end-stage prpTDP-43^{A315T} mice. Scale bars, 250 μm. (**D**) High magnification representative merged (first panel) images of UMNs (second panel) and B8H10 antibody staining (third panel) that recognizes misfolded SOD1 protein in the motor cortex of untreated WT-UeGFP or (**E**) untreated end-stage hSOD1^{G93A}-UeGFP mice or (**F**) untreated end-stage prpTDP-43^{A315T} mice. Scale bars, 20 μm.

Table S1: Patient information

Gender	Age of onset	Age of death	Clinical Diagnosis and Pathological assessment	TDP+ NCIs	TDP + GCIs	TDP + DNs	PMI/h
M	59	61	sALS with TDP-43	+	0	0	29
M	53	61	sALS with TDP-43	+	0	0	13
M	71	73	sALS with TDP-43	0	+	0	23
M	61	64	sALS with TDP-43	0	+	0	14
M	59	64	sALS with TDP-43	+	++	+	19
M	37	40	sALS with TDP-43	+	++	+	19
M	55	57	fALS with TDP-43	+	+++	0	18
F	78	82	fALS with TDP-43	++	0	0	36
F	62	64	sALS with TDP-43	+	+	0	19
M	N/A	54	Normal control	0	0	0	12
M	N/A	72	Normal control	0	0	0	14
F	N/A	45	Normal control	0	0	0	15
F	N/A	78	Normal control	0	0	0	8

Table S2: Mice included in *in vivo* studies.

**Genotype**	**Treatment**	**Number of mice**
WT-UeGFP	vehicle	10
WT-UeGFP	**NU-9** (20 mg/kg/day)	5
WT-UeGFP	**NU-9** (100 mg/kg/day)	11
hSOD1^{G93A}-UeGFP	vehicle	6
hSOD1^{G93A}-UeGFP	**NU-9** (20 mg/kg/day)	7
hSOD1^{G93A}-UeGFP	**NU-9** (100 mg/kg/day)	9
prpTDP-43^{A315T}-UeGFP	untreated	3
prpTDP-43^{A315T}-UeGFP	**NU-9** (100 mg/kg/day)	4

Table S4: Number of mice, total number of UMN, total number of mitochondria, and total number of ER cistarnae used for electron microscopy analysis.

**Genotype**	**WT-UeGFP**	**hSOD1^{G93A}-UeGFP treated with vehicle**	**hSOD1^{G93A}-UeGFP treated with NU-9***	**prpTDP-43^{A315T}-UeGFP untreated**	**prpTDP-43^{A315T}-UeGFP treated with NU-9***
Number of mice	4	4	4	3	4
Total number of cells counted	55	46	46	37	41
Total number of mitochondria counted	1647	401	966	748	1306
Total number of ER cisternae counted	1550	161	646	421	1395

* **NU-9** treatment: 100 mg/kg/day dose by gavage.
